# Microbial transformations of 4′-methylchalcones as an efficient method of obtaining novel alcohol and dihydrochalcone derivatives with antimicrobial activity†

**DOI:** 10.1039/c8ra04669g

**Published:** 2018-08-30

**Authors:** Joanna Kozłowska, Bartłomiej Potaniec, Barbara Żarowska, Mirosław Anioł

**Affiliations:** Department of Chemistry, Wrocław University of Environmental and Life Sciences Norwida 25 50-375 Wrocław Poland joannakozlowska3@gmail.com; Department of Biotechnology and Food Microbiology, Wrocław University of Environmental and Life Sciences Chełmońskiego 37 51-630 Wrocław Poland

## Abstract

Biotransformations are an alternative method of receiving dihydrochalcones as a result of the reduction of α,β-unsaturated ketones – chalcones. In presented research, two strains of bacteria – *Gordonia* sp. DSM44456 and *Rhodococcus* sp. DSM364 – were selected as effective biocatalysts that are able to transform chalcones in a short period of time. As a result of our investigation 3 new dihydrochalcones and one novel alcohol were obtained with high isolated yields. All 4′-methylchalcone derivatives and biotransformations products were tested for antimicrobial activity against *Escherichia coli* ATCC10536, *Staphylococcus aureus* DSM799, *Candida albicans* DSM1386, *Alternaria alternata* CBS1526, *Fusarium linii* KB-F1, and *Aspergillus niger* DSM1957. The best inhibitory effect was observed for all chalcones against *E. coli* ATCC10536 – compounds 1–6 and 8 prevented thorough growth of this strain (ΔOD = 0). Moreover, dihydrochalcones showed about 2–3 times stronger inhibitory effect against *S. aureus* DSM799 in comparison to their chalcones. Excluding the *E. coli* ATCC10536 strain, 3-(4-carboxyphenyl)-1-(4-methylphenyl)propan-1-ol (8b) had weaker biological activity than 4-carboxy-4′-methyl-α,β-dihydrochalcone (8a).

## Abstract 2

Biotransformations of 4′-methylchalcones using whole cells of bacteria allowed to obtain dihydrochalcones and alcohol with high isolated yields. The antimicrobial activity of all derivatives was evaluated against selected bacteria and fungi strains.

## Graphical abstract

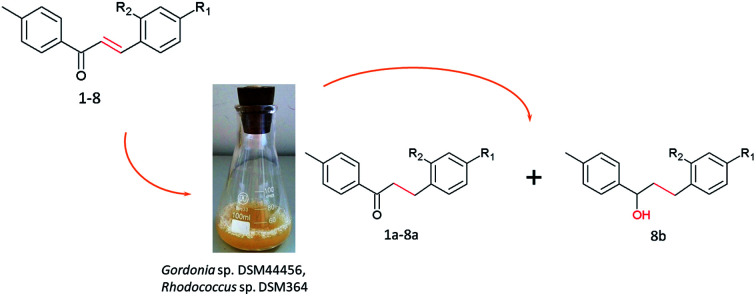

## Introduction

Chalcones (1,3-diaryl-2-propen-1-ones) belong to a wide group of polyphenols known as flavonoids. Their chemical structure is characterized by the α,β-unsaturated bond formed as the result of opening ring C in flavanones.^1^ The presence of numerous electron-withdrawing or donating groups allows to describe chalcones as compounds with various biological activities such as anti-inflammatory,^2^ antioxidant,^3^ antibacterial^4^ and antitumor.^5,6^

Hydrogenation of the α,β-unsaturated bond in chalcones leads to derivatives called dihydrochalcones. Interestingly, neohesperidin occurring in citrus fruits is characterized with a bitter taste in contrast to the neohesperidin dihydrochalcone – hypocaloric sweetener accepted by the EU and FDA as a food additive.^7^ Furthermore, antioxidant properties of dihydrochalcones were observed to be significantly stronger than corresponding flavanones.^8^ Moreover, many potential anticancer agents have already been found among chalcones. For example, 2′,6′-dihydroxy-4′4-dimethoxydihydrochalcone, 2′,6′-dihydroxy-4′-methoxydihydrochalcone and 2′,4′,6′-trihydroxydihydrochalcone (phloretin) augmented apoptosis in prostate cancer cells.^9^

According to literature, the chemoselective reduction of the α,β-unsaturated bond is the result of hazardous reagents – some of them requiring transition metals for reaction.^10,11^ Microbial transformations are a good alternative to chemical synthesis, transforming chalcones into dihydrochalcones without using harmful and toxic compounds. Also, the application of whole cells of microorganisms is often superior to enzymatic modifications, that usually are more costly, and need additional cofactors and strict incubation conditions. Moreover, multistep reactions, impeded by the traditional approach of chemical synthesis, are possible with the enzymatic apparatus of microbials.

The most frequently described modifications of flavonoids catalysed by microbial cells are hydroxylation, dehydroxylation, *O*-methylation, *O*-demethylation, glycosylation, deglycosylation, dehydrogenation, hydrogenation, C ring cleavage, cyclization and reduction.^12^ Enoate reductase has been characterised as one of the main enzymes responsible for reduction carbon–carbon double bonds. It belongs to the Old Yellow Enzyme (OYE) family, consisting of the flavin-dependent oxidoreductases, that require NADH to be active.^13^ Current knowledge proofs the presence of enoate reductases in known biocatalysts such as bacteria and fungi strains.^14,15^ Additionally, recent a report described the bioconversion of *trans*-chalcone by whole cells of cyanobacteria.^16^ This variety of active whole-cell catalysts creates a possibility of obtaining novel derivatives with enhanced biological properties. In recent years, antimicrobial activity of flavonoids has gained much interest.

Infections caused by microorganisms are one of the most important problems in all aspects of our life, especially in schools, hospitals and workplaces. Increasing resistance of microorganisms to commonly used antibiotics encourages the exploration of novel active compounds, often from natural sources. The variety of chalcones and dihydrochalcones derivatives can hinder bacteria growth, including multi-resistant strains.^4,17^ Phloretin and their glycosylated derivatives – phlorizin and phloretin 3′,5′-di-C-glucoside – are known growth inhibitors of Gram-positive bacteria such as *Staphylococcus aureus* ATCC6538, clinical strains of methicillin-resistant *Staphylococcus aureus* (MRSA) and *Listeria monocytogenes* ATCC13932. Furthermore, phloretin showed antibacterial activity against Gram-negative bacteria – *Salmonella typhimurium* ATCC13311.^18^ Also, the investigation performed by a scientific group in Brazil showed that 4-methoxy-4′-methylchalcone (3) hindered the growth of Gram-positive bacteria *Mycobacterium tuberculosis* H37Rv with a MIC_50_ value of 66.9 μM.^19^

In our paper, we describe microbial transformation of series of 4′-methylchalcone derivatives (1–8) performed by two species of aerobic bacteria – *Gordonia* sp. DSM44456 and *Rhodococcus* sp. DSM364. Previous experiments carried out by our research team allowed us to select two strains of bacteria capable of transforming chalcones to dihydrochalcones with high isolated yields.^14^ Moreover, we observed that elongation of biotransformation time provides a second metabolite: the alcohol. Subsequently, an evaluation of antimicrobial activity against six strains of microorganisms *Escherichia coli* ATCC10536, *Staphylococcus aureus* DSM799, *Candida albicans* DSM1386, *Alternaria alternata* CBS1526, *Fusarium linii* KB-F1, and *Aspergillus niger* DSM1957 was prepared for synthetized chalcones (1–8) and compared with their biotransformation products (1a–8a, 8b).

## Experimental

### Materials

All 4′-methylchalcone derivatives (1–8) were prepared with the procedure described by Amir *et al.* with yield 60–94%.^20^ Biotransformations were performed using two strains of bacteria – *Gordonia* sp. DSM44456 and *Rhodococcus* sp. DSM364. Strains were obtained from the German Collection of Microorganisms and Cell Cultures GmbH (DSMZ, Deutsche Sammlung von Mikroorganismen und Zellkulturen, GmbH).

### Biotransformation in small scale

Screening procedure was performed in Erlenmeyer flasks (100 mL) containing 25 mL of culture medium, which consisted of 10 g peptone, 2 g casein hydrolysate, 2 g yeast extract, 6 g sodium chloride and 20 g glucose dissolved in 1000 mL distilled water. Sterile culture medium was inoculated using 0.1 mL of bacteria suspension and kept at 28 °C on rotary shaker. After 48 hours, 1 mg of 4′-methylchalcone derivative dissolved in 0.5 mL acetone (1–7) or dimethyl sulfoxide (DMSO) (8) was added to the culture. Progress of biotransformation was monitored by the thin layer chromatography (TLC) and the liquid high-performance chromatography (HPLC).

### Biotransformation in preparative scale

To isolate products of biotransformation observed in small scale, preparative investigation was performed using 2000 mL Erlenmeyer flasks containing 300 mL of sterile culture medium, inoculated with 1 mL of bacteria suspension and maintained at 28 °C on rotary shaker. After 48 hours 30 mg of 4′-methylchalcone derivative dissolved in 1 mL acetone (1–7) or DMSO (8) was added to the grown culture and incubation under the same conditions was continued. After determining the complete substrate conversion, biotransformation mixture was extracted with ethyl acetate (3 × 100 mL). Organic fractions were collected and dried over anhydrous sodium sulphate. Organic solvent was evaporated on vacuum evaporator and biotransformation extract was purified on column chromatography. Purity of obtained products was verified by HPLC and structures were determined using ^1^H and ^13^C NMR.

### Analytical methods

Progress of biotransformations performed in small and preparative scale was analysed by thin layer chromatography (TLC) on silica gel-coated aluminium plates with fluorescent indicator (DC-Alufolien, Kieselgel 60 F_254_; Merck, Darmstadt, Germany) using mixture of hexane : ethyl acetate or chloroform : methanol as eluents. Products were detected by spraying the plates with a solution of 1% Ce(SO_4_)_2_ and 2% H_3_[P(Mo_3_O_10_)_4_] in 5% H_2_SO_4_ and subsequently visualised by heating. Extracts obtained from preparative biotransformations were purified by liquid column chromatography using silica gel (Kieselgel 60, 230–400 mesh, Merck). The purity of the products was analysed by HPLC on a Waters 2690 (Milford, MA, USA) with Photodiode Array Detector Waters 996. The samples of substrates and biotransformation products for HPLC analysis were dissolved in methanol. The HPLC apparatus was equipped with a reverse-phase C-18 column (Phenomenex, Torrance, CA, United States, Kinetex 5u XB-C18 100A, 250 mm × 4.6 mm), which was thermostated at 28 °C, and analysed samples were kept at 12 °C. The mobile phase consisted of two eluents: A—1% HCOOH in MeCN and B—1% HCOOH in H_2_O. Elution gradient was started from 55% of eluent A to 45% of eluent B over 15 min. A flow rate of 1.5 mL min^−1^ was used.

The structure of the obtained compounds was confirmed using nuclear magnetic resonance (NMR) analysis. ^1^H-NMR and ^13^C-NMR spectra were recorded on a Bruker Avance™600 MHz spectrometer (Bruker, Billerica, MA, USA) with chloroform-d as solvent.

Positive and negative-ion HR ESI-MS spectra were measured on a Bruker ESI-Q-TOF Maxis Impact Mass Spectrometer (Bruker, Billerica, MA, USA). The direct infusion of ESI-MS parameters: the mass spectrometer was operated in positive (1a–8a) and negative (8b) ion mode with the potential of 3.5 kV between the spray needle and the orifice, nebulizer pressure of 0.4 bar, and a drying gas flow rate of 3.0 L min^−1^ at 200 °C. The sample flow rate was set as 3.0 μL min^−1^. Ionization mass spectra were collected at the ranges *m*/*z* 50–1250.

Infrared spectra were determined using a Nicolet 6700 FTIR spectrometer (Thermo Scientific, Waltham, MA, USA) with ATR accessory with diamond crystal in the wavelength range 400–4000 cm^−1^.

Optical rotation was measured on Jasco P-2000 automatic polarimeter (ABL&E-JASCO, Kraków, Poland). Solution was prepared in chloroform and the concentration was expressed in g/100 mL.

### Biological assays

To evaluate activity of 4′-methylchalcones and their derivatives obtained as a result of biotransformations, a series of antimicrobial assays were performed. In our investigation two strains of bacteria: *E. coli* ATCC10536 and *S. aureus* DSM799 and four strains of fungi: *C. albicans* DSM1386, *F. linii* KB-F1, *A. alternata* CBS1526 and *A. niger* DSM1957 were used. All the microorganisms were obtained from the collection of the Faculty of Biotechnology and Food Microbiology, Wroclaw University of Environmental and Life Sciences. Bacteria were cultured in the standard nutrient broth (Biocorp, Warsaw, Poland), and fungi in the YM medium, which consisted of 3 g yeast extract, 3 g malt extract, 5 g bacteriological peptone and 10 g of glucose dissolved in 1 L of distilled water. Assays were performed on 100-well microtiter plates, with the working volume of 300 μL per well: 280 μL of culture medium, 10 μL of microorganism suspension and 10 μL of 4′-methylchalcone or its derivative dissolved in DMSO (0.3% (w/v)). The final concentration of the tested substance was 0.1% (w/v). Each culture was prepared in triplicate. The optical density (OD) of the cell suspensions was measured on Bioscreen C (Automated Growth Curve Analysis System Lab System, Helsinki, Finland) at 560 nm automatically, at regular intervals of 30 min for 2–3 days. Cell cultures were maintained at 28 °C on a continuous shaker. The growth curves for each strain were prepared as a mean values of the measured OD as a function of time. The resulting antimicrobial activity was expressed as the increase in optical density (ΔOD) in comparison to the control cultures in the medium supplemented with dimethyl sulfoxide.

## Results and discussion

### Biotransformations

Chalcones received in classical Claisen-Schmidt reaction were used as a substrate to biotransformation.

**Scheme 1:**
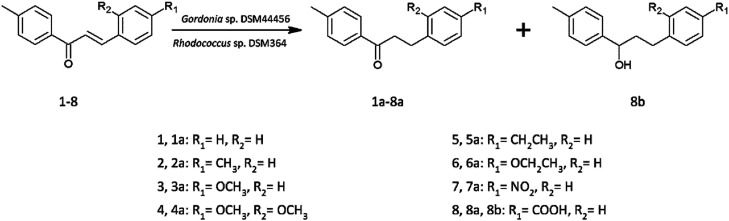
Biotransformations of 4′-methylchalcone derivatives in *Gordonia* sp. DSM44456 and *Rhodococcus* sp. DSM364 cultures.

**Scheme 2:**
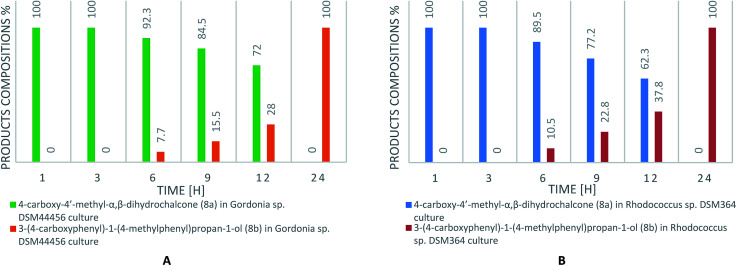
The content of biotransformation products (8a, 8b) of 4-carboxy-4′-methylchalcone (8) in *Gordonia* sp. DSM44456 (A) and *Rhodococcus* sp. DSM364 (B) cultures (according to HPLC).

Current literature describes the biotransformation of 4′-methylchalcone (1) to dihydrochalcone (1a) in *Corynebacterium equi* IFO 3730 culture after 3 days with 94% yield.^21^ However, our investigation have showed complete conversion of all substrates (1–8) already after maximum 24 hours with isolated yields up to 99%. Microbial transformations were monitored in time using thin layer chromatography and high-performance liquid chromatography. After the disappearance of the substrate, extraction and purification on liquid chromatography gave us pure product. The structure of all the compounds was determined by nuclear magnetic resonance analysis. Biotransformations of substrates 1–7 resulted in obtaining their dihydrochalcones (Scheme 1). Interestingly, the product of 4-carboxy-4′-methylchalcone (8) transformation under the same cultivation condition appeared to be the alcohol (8b), not the expected dihydrochalcone. The optical rotation of novel 3-(4-carboxyphenyl)-1-(4-methylphenyl)propan-1-ol (8b) was measured in chloroform and the value [*α*]^20^_D_ = −2.733 (*c* = 0.9) was obtained. This result proved, that product of biotransformation was a mixture of isomers with a predominance of one of them. The rapidity of reduction led us to analyse the biotransformation process in shorter intervals of time (after 1, 3, 6, 9, 12 and 24 hours). The results are shown in Scheme 2. After 1 hour of biotransformation of 4-carboxy-4′-methylchalcone (8), the complete conversion to compound 8a by both strains was observed. Furthermore, after 6 hours of microbial transformation the second product (8b) was observed. In addition, the conversion in *Rhodococcus* sp. DSM364 culture was faster in comparison to *Gordonia* sp. DSM44456. Both products of the biotransformation of 4-carboxy-4′-methylchalcone (8) were isolated. The NMR analysis confirmed our supposition that the product observed after 1 hour was 4-carboxy-4′-methyl-α,β-dihydrochalcone (8a). The presence of electron withdrawing group (*e.g.* carboxylic, nitro group) usually enhance the rate of hydrogenation catalyzed by enoate reductase.^22^ However, accelerated process of biotransformation was observed only in the case of 4-carboxy-4′-methylchalcone (8). Most likely, the effect of action was connected with better solubility of substrate or uptake by microorganism cells caused by presence of carboxylic group.^23^

The efficiency of microbial transformation in *Gordonia* sp. DSM44456 and *Rhodococcus* sp. DSM364 culture described Table 1.

**Table 1:** Isolated yields (%) of biotransformation products in *Gordonia* sp. DSM44456 and *Rhodococcus* sp. DSM364 culture (compounds 1a–7a and 8b isolated after 24 h, compound 8a isolated after 1 h)

Product	*Gordonia* sp.	*Rhodococcus* sp.
1a	62.9	77.5
2a	55.0	50.7
3a	84.1	82.6
4a	80.2	96.4
5a	37.7	40.3
6a	43.3	40.6
7a	79.0	70.9
8a	86.0	99.1
8b	71.9	82.0

### Spectral data of biotransformation products

The structures of biotransformation products were determined by ^1^H-NMR, ^13^C-NMR and HR ESI-MS analysis. Additionally, FTIR-ATR spectra of novel compounds were recorded. All data are described in details below.

4′-Methyl-α,β-dihydrochalcone (1a), white solid, mp 61–63 °C (lit.^24^ 61–63 °C); ^1^H NMR (600 MHz, CDCl_3_): *δ* 7.90–7.83 (2H, m, H-2′, H-6′, AA′BB′), 7.33–7.24 (6H, m, H-2, H-3, H-5, H-6, H-3′, H-5′), 7.24–7.19 (1H, m, H-4), 3.32–3.25 (2H, m, H-α), 3.11–3.02 (2H, m, H-β), 2.41 (3H, s, C-4′-C
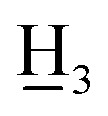
); ^13^C NMR (150 MHz, CDCl_3_): *δ* 199.00 (C̲=O), 143.94 (C-4′), 141.52 (C-1), 134.51 (C-1′), 129.39 (C-2′, C-6′), 128.63 (C-3, C-5), 128.54 (C-2, C-6), 128.28 (C-3′, C-5′), 126.21 (C-4), 40.47 (C-α), 30.34 (C-β), 21.76 (C-4′-C̲H_3_); HR ESI-MS *m*/*z* calculated for C_16_H_17_O [M + H]^+^ 225.1274, found [M + H]^+^ 225.1282.

4′-Methyl-4-methyl-α,β-dihydrochalcone (2a), white solid, mp 58–60 °C (lit.^25^ 59–61 °C); ^1^H NMR (600 MHz, CDCl_3_): *δ* 7.88–7.84 (2H, m, H-2′, H-6′, AA′BB′), 7.25 (2H, d, *J* = 8.7 Hz, H-3′, H-5′), 7.15 (2H, d, *J* = 7.9 Hz, H-3, H-5), 7.12 (2H, d, *J* = 7.9 Hz, H-2, H-6), 3.29–3.22 (2H, m, H-α), 3.06–2.99 (2H, m, H-β), 2.41 (3H, s, C-4′-C
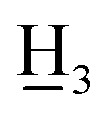
), 2.33 (3H, s, C-4-C
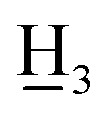
); ^13^C NMR (150 MHz, CDCl_3_): *δ* 199.14 (C̲=O), 143.91 (C-4′), 138.42 (C-4), 135.70 (C-1), 134.54 (C-1′), 129.39 (C-3, C-5), 129.31 (C-3′, C-5′), 128.42 (C-2, C-6), 128.29 (C-2′, C-6′), 40.65 (C-α), 29.94 (C-β), 21.77 (C-4′-C̲H_3_), 21.14 (C-4-C̲H_3_); HR ESI-MS *m*/*z* calculated for C_17_H_19_O [M + H]^+^ 239.1430, found [M + H]^+^ 239.1435.

4-Methoxy-4′-methyl-α,β-dihydrochalcone (3a), white solid, mp 54–55 °C (lit.^26^ 57–58 °C); ^1^H NMR (600 MHz, CDCl_3_): *δ* 7.89–7.82 (2H, m, H-2′, H-6′, AA′BB′), 7.29–7.19 (2H, m, H-3′, H-5′, AA′BB′), 7.21–7.12 (2H, m, H-2, H-6, AA′BB′), 6.87–6.81 (2H, m, H-3, H-5, AA′BB′), 3.79 (3H, s, C-4-OC
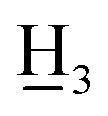
), 3.28–3.20 (2H, m, H-α), 3.04–2.96 (2H, m, H-β), 2.41 (3H, s, C-4′-C
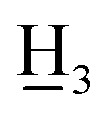
); ^13^C NMR (150 MHz, CDCl_3_): *δ* 199.17 (C̲=O), 158.08 (C-4), 143.90 (C-4′), 134.56 (C-1′), 133.54 (C-1), 129.46 (C-2, C-6), 129.39 (C-2′, C-6′), 128.29 (C-3′, C-5′), 114.04 (C-3, C-5), 55.40 (C-4-OC̲H_3_), 40.73 (C-α), 29.50 (C-β), 21.75 (C-4′-C̲H_3_); HR ESI-MS *m*/*z* calculated for C_17_H_19_O_2_ [M + H]^+^ 255.1380, found [M + H]^+^ 255.1381.

2,4-Dimethoxy-4′-methyl-α,β-dihydrochalcone (4a), yellow oil; FTIR-ATR (cm^−1^): 2930.60, 2835.33, 1678.26, 1607.03, 1587.71, 1506.01, 1456.13, 1290.57, 1205.54, 1179.77, 1150.03, 1035.53, 824.73; ^1^H NMR (600 MHz, CDCl_3_): *δ* 7.92–7.80 (2H, m, H-2′, H-6′, AA′BB′), 7.30–7.19 (2H, m, H-3′, H-5′, AA′BB′), 7.09 (1H, d, *J* = 8.2 Hz, H-6), 6.46 (1H, d, *J* = 2.5 Hz, H-3), 6.42 (1H, dd, *J* = 8.2, 2.5 Hz, H-5), 3.80 (3H, s, C-2-OC
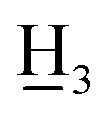
), 3.79 (3H, s, C-4-OC
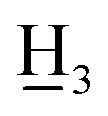
), 3.23–3.09 (2H, m, H-α), 3.02–2.87 (2H, m, H-β), 2.40 (3H, s, C-4′-C
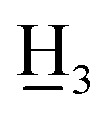
); ^13^C NMR (150 MHz, CDCl_3_): *δ* 199.97 (C̲=O), 159.57 (C-4), 158.49 (C-2), 143.69 (C-4′), 134.67 (C-1′), 130.43 (C-6), 129.31 (C-3′, C-5′), 128.37 (C-2′, C-6′), 122.12 (C-1), 103,95 (C-5), 98.67 (C-3), 55.50 (C-2-OC̲H_3_), 55.35 (C-4-OC̲H_3_), 39.26 (C-α), 25.34 (C-β), 21.76 (C-4′-C̲H_3_); HR ESI-MS *m*/*z* calculated for C_18_H_21_O_3_ [M + H]^+^ 285.1485, found [M + H]^+^ 285.1486.

4-Ethyl-4′-methyl-α,β-dihydrochalcone (5a), white solid, mp 36–38 °C; FTIR-ATR (cm^−1^): 2971.60, 2921.82, 1679.29, 1604.56, 1574.48, 1514.73, 1441.94, 1406.62, 1360.86, 1289.24, 1201.94, 1184.31, 1059.99, 990.29, 971.67, 819.19, 767.02; ^1^H NMR (600 MHz, CDCl_3_): *δ* 7.88 (2H, d, *J* = 8.2 Hz, H-2′, H-6′), 7.26 (2H, d, *J* = 8.2 Hz, H-3′, H-5′), 7.19 (2H, d, *J* = 8.1 Hz, H-2, H-6), 7.15 (2H, d, *J* = 8.1 Hz, H-3, H-5), 3.30–3.24 (2H, m, H-α), 3.08–3.01 (2H, m, H-β), 2.64 (2H, q, *J* = 7.6 Hz, –C
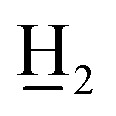
CH_3_), 2.42 (3H, s, C-4′-C
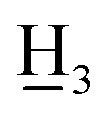
), 1.24 (3H, t, *J* = 7.6 Hz, –CH_2_C
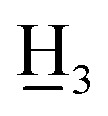
); ^13^C NMR (150 MHz, CDCl_3_): *δ* 199.12 (C̲=O), 143.88 (C-4′), 142.12 (C-4), 138.67 (C-1), 134.54 (C-1′), 129.37 (C-3′, C-5′), 128.46 (C-2, C-6), 128.28 (C-2′, C-6′), 128.10 (C-3, C-5), 40.60 (C-α), 29.94 (C-β), 28.57 (–C̲H_2_CH_3_), 21.74 (C-4′-C̲H_3_), 15.78 (–CH_2_C̲H_3_); HR ESI-MS *m*/*z* calculated for C_18_H_21_O [M + H]^+^ 253.1587, found [M + H]^+^ 253.1588.

4-Ethoxy-4′-methyl-α,β-dihydrochalcone (6a), white solid, mp 68–70 °C; ^1^H NMR (600 MHz, CDCl_3_): *δ* 7.89–7.83 (2H, m, H-2′, H-6′, AA′BB′), 7.26–7.23 (2H, m, H-3′, H-5′, AA′BB′), 7.18–7.13 (2H, m, H-2, H-6, AA′BB′), 6.86–6.80 (2H, m, H-3, H-5, AA′BB′), 4.01 (2H, q, *J* = 7.0 Hz, –OC
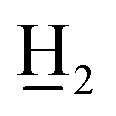
CH_3_), 3.27–3.21 (2H, m, H-α), 3.03–2.97 (2H, m, H-β), 2.41 (3H, s, C-4′-C
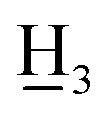
), 1.40 (3H, t, *J* = 7.0 Hz, –OCH_2_C
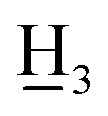
); ^13^C NMR (150 MHz, CDCl_3_): *δ* 199.22 (C̲=O), 157.44 (C-4), 143.90 (C-4′), 134.57 (C-1′), 133.41 (C-1), 129.44 (C-2, C-6), 129.39 (C-2′, C-6′), 128.30 (C-3′, C-5′), 114.66 (C-3, C-5), 63.56 (–OC̲H_2_CH_3_), 40.75 (C-α), 29.53 (C-β), 21.76 (C-4′-C̲H_3_), 15.02 (–OCH_2_C̲H_3_); HR ESI-MS *m*/*z* calculated for C_18_H_21_O_2_ [M + H]^+^ 269.1536, found [M + H]^+^ 269.1539.

4′-Methyl-4-nitro-α,β-dihydrochalcone (7a), white solid, mp97–100 °C (lit.^27^ 104–105 °C); ^1^H NMR (600 MHz, CDCl_3_): *δ* 8.17–8.12 (2H, m, H-3, H-5, AA′BB′), 7.87–7.82 (2H, m, H-2′, H-6′, AA′BB′), 7.44–7.39 (2H, m, H-2, H-6, AA′BB′), 7.26 (2H, d, *J* = 8.5, H-3′, H-5′), 3.32 (2H, t, *J* = 7.4 Hz, H-α), 3.18 (2H, t, *J* = 7.4 Hz, H-β), 2.41 (3H, s, C-4′-C
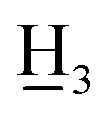
); ^13^C NMR (150 MHz, CDCl_3_): *δ* 197.91 (C=O), 149.45 (C-4), 146.63 (C-4′), 144.39 (C-1), 134.18 (C-1′), 129.52 (C-2, C-6), 129.49 (C-3′, C-5′), 128.24 (C-2′, C-6′), 123.88 (C-3, C-5), 39.41 (C-α), 29.96 (C-β), 21.80 (C-4′-C̲H_3_); HR ESI-MS *m*/*z* calculated for C_16_H_16_NO_3_ [M + H]^+^ 270.1125, found [M + H]^+^ 270.1128.

4-Carboxy-4′-methyl-α,β-dihydrochalcone (8a), white solid, mp 142–145 °C; FTIR-ATR (cm^−1^): 2920.80, 2850.12, 1676.92, 1608.75, 1574.89, 1426.10, 1403.52, 1315.66, 1291.75, 1236.27, 1173.49, 1101.72, 1043.14, 972.35, 931.83, 811.41, 769.06; ^1^H NMR (600 MHz, CDCl_3_): *δ* 8.04 (2H, d, *J* = 8.2 Hz, H-3, H-5), 7.86 (2H, d, *J* = 8.2 Hz, H-2′, H-6′), 7.35 (2H, d, *J* = 8.2 Hz, H-2, H-6), 7.25 (2H, d, *J* = 8.2 Hz, H-3′, H-5′), 3.31 (2H, t, *J* = 7.6 Hz, H-α), 3.14 (2H, t, *J* = 7.6 Hz, H-β), 2.41 (3H, s, C-4′-C
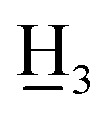
); ^13^C NMR (150 MHz, CDCl_3_): *δ* 198.48 (C̲=O), 172.01 (C̲OOH), 148.03 (C-4′), 144.20 (C-1), 134.33 (C-1′), 130.63 (C-3, C-5), 129.47 (C-3′, C-5′), 128.75 (C-2, C-6), 128.29 (C-2′, C-6′), 127.47 (C-4), 39.78 (C-α), 30.31 (C-β), 21.79 (C-4′-C̲H_3_); HR ESI-MS *m*/*z* calculated for C_17_H_17_O_3_ [M + H]^+^ 269.1172, found [M + H]^+^ 269.1179.

3-(4-Carboxyphenyl)-1-(4-methylphenyl)propan-1-ol (8b) white solid, mp 134–137 °C; [*α*]^20^_D_= −2.733 (*c* = 0.9; CHCl_3_); FTIR-ATR (cm^−1^): 3541.71, 2928.77, 2852.96, 1681.48, 1610.44, 1575.23, 1512.98, 1423.63, 1314.93, 1289.47, 1174.00, 1063.42, 925.05, 814.95, 765.85; ^1^H NMR (600 MHz, CDCl_3_): *δ* 8.01 (2H, d, *J* = 8.2 Hz, H-3, H-5), 7.28 (2H, d, *J* = 7.9 Hz, H-2, H-6), 7.24 (2H, d, *J* = 7.9 Hz, H-2′, H-6′), 7.17 (2H, d, *J* = 7.8 Hz, H-3′, H-5′), 4.65 (1H, dd, *J* = 7.8, 5.4 Hz, –CH̲–OH), 2.84–2.77 (1H, m, H-β), 2.77–2.69 (1H, m, H-β), 2.35 (3H, s, C-4′-C
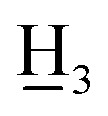
), 2.19–2.11 (1H, m, H-α), 2.07–1.98 (1H, m, H-α); ^13^C NMR (150 MHz, CDCl_3_): *δ* 171.83 (C̲OOH), 148.54 (C-1′), 141.39 (C-1), 137.67 (C-4′), 130.50 (C-3, C-5), 129.41 (C-3′, C-5′), 128.74 (C-2, C-6), 127.24 (C-4), 126.00 (C-2′, C-6′), 73.75 (–C̲H–OH), 40.04 (C-α), 32.34 (C-β), 21.27 (C-4′-C̲H_3_); HR ESI-MS *m*/*z* calculated for C_17_H_17_O_3_ [M − H]^−^ 269.1172, found [M − H]^−^ 269.1178.

### Antimicrobial activity

Broad spectrum of biological properties of chalcones and their derivatives is associated with the diversity of these polyphenolic compounds. Chalcones and dihydrochalcones belong to secondary plant metabolites and constitute a natural protective barrier against microbial infections. Phloretin is one of the most popular dihydrochalcones exhibiting strong antimicrobial activity against *Erwinia amylovora* and *Venturia inaequalis* – Gram-negative bacteria and fungus, which are a common cause of apple diseases.^28^

As a result of our investigations, antimicrobial activity of synthesised chalcones (1–8) and biotransformation products (1a–8a, 8b) was characterized. Inhibitory effect of all compounds was evaluated against two strains of bacteria and four strains of fungi (Tables 2 and 3).

**Table 2:** Antimicrobial activity of 4′-methylchalcones (1–8)^a^

Strain		*E. coli* ATCC10536	*S. aureus* DSM799	*C. albicans* DSM1386	*A. alternata* CBS1526	*F.linii* KB-F1	*A. niger* DSM1957
Control	Lag-phase [h]	4.0	3.0	3.0	16.5	13.5	10.5
	ΔOD	1.62	1.66	1.56	1.85	1.93	2.13
1	Lag-phase [h]	—	23.5	5.0	26.0	3.5	27.0
	ΔOD	0.0	1.42	1.54	1.03	1.34	0.91
2	Lag-phase [h]	—	26.0	6.0	19.0	2.5	33.5
	ΔOD	0.0	1.22	1.53	0.83	0.96	0.70
3	Lag-phase [h]	—	24.0	4.5	18.0	2.5	20.5
	ΔOD	0.0	1.08	1.32	0.82	1.14	0.69
4	Lag-phase [h]	—	5.0	7.5	36.5	9.0	35.5
	ΔOD	0.0	0.46	0.63	0.23	0.75	0.28
5	Lag-phase [h]	—	24.5	5.0	20.0	3.5	20.5
	ΔOD	0.0	1.30	1.63	1.06	1.33	1.12
6	Lag-phase [h]	—	24.0	5.0	19.5	3.0	20.5
	ΔOD	0.0	1.04	1.19	0.68	1.03	0.74
7	Lag-phase [h]	22.0	26.0	6.5	34.0	6.0	34.0
	ΔOD	0.31	1.02	1.02	0.93	0.93	1.08
8	Lag-phase [h]	—	28.5	16.5	—	3.5	—
	ΔOD	0.0	0.45	0.29	0.0	0.32	0.0

a OD – optical density (*λ* = 560 nm).

**Table 3:** Antimicrobial activity of products of biotransformations (1a–8a, 8b)^a^

Strain		*E. coli* ATCC10536	*S. aureus* DSM799	*C. albicans* DSM1386	*A. alternata* CBS1526	*F.linii* KB-F1	*A. niger* DSM1957
Control	Lag-phase [h]	4.0	2.5	3.0	16.5	13.5	11.0
	ΔOD	1.61	1.67	1.58	1.86	1.95	2.14
1a	Lag-phase [h]	16.5	4.0	8.0	18.0	14.5	29.0
	ΔOD	0.17	0.54	0.61	0.74	1.21	0.77
2a	Lag-phase [h]	15.5	7.5	5.0	16.5	9.5	30.0
	ΔOD	0.15	0.56	0.78	1.20	1.08	0.92
3a	Lag-phase [h]	15.5	6.0	8.0	18.5	14.5	29.0
	ΔOD	0.24	0.51	0.69	1.07	1.19	0.63
4a	Lag-phase [h]	15.0	7.5	8.5	17.0	13.0	31.5
	ΔOD	0.31	0.48	0.61	0.96	1.21	0.99
5a	Lag-phase [h]	15.5	8.0	12.0	25.0	13.0	31.5
	ΔOD	0.30	0.43	0.74	1.49	1.27	0.97
6a	Lag-phase [h]	4.5	2.5	7.0	18.0	12.0	28.0
	ΔOD	0.44	0.63	0.82	1.28	1.17	0.89
7a	Lag-phase [h]	14.5	6.0	8.0	17.5	10.0	35.5
	ΔOD	0.10	0.48	0.53	0.77	0.94	0.94
8a	Lag-phase [h]	14.0	18.0	14.0	24.0	11.0	—
	ΔOD	0.10	0.19	0.30	0.34	0.24	0.0
8b	Lag-phase [h]	—	10.5	21.0	18.5	21.0	35.0
	ΔOD	0.0	0.45	0.64	0.51	0.67	0.91

a OD – optical density (*λ* = 560 nm).

Previously, Stompor *et al.* tested antibacterial activity of 4′-methoxychalcone derivatives against *E. coli* PCM2560, *S. aureus* PCM2054 and *C. albicans* KL-1 using agar diffusion method with 10 μL of 20% (m/v) compounds applied onto each test disc. They reported, that chalcones with methoxy group attached to the C-4′ position had no bacteriostatic effect on *E. coli* PCM2560.^29^ As a result of our study, complete inhibition of growth of *E. coli* ATCC10536 in presence of compounds 1–6 and 8 was observed. Contrarily, 4′-methyl-4-nitrochalcone (7) enabled the limited growth of this strain of bacteria (ΔOD = 0.31) and additionally caused prolongation of the lag phase from 4 to 22 hours. Sivakumar *et al.* calculated the Minimal Inhibitory Concentration (MIC) of 4-methoxy-4′-methylchalcone against *S. aureus* NCIM5021 at the level of 0.248 μM. Unfortunately, this activity is three times weaker than the popular antibiotic ampicillin.^30^ In our research, 4′-methylchalcone derivatives with alkyl, *O*-alkyl or nitro group attached to the C-4 position, also exhibited a very weak effect considering limitation of *S. aureus* DSM799 growth, but they prolonged the adaptive phase from 3 to 23.5–26.0 hours. However, 2,4-dimethoxy-4′-methylchalcone (4) exhibited activity that was twice as strong as the chalcone without methoxy group attached to the C-2 position (3). A similar effect was observed for 4-carboxy-4′-methylchalcone, which also extended the lag phase of *S. aureus* DSM799. López *et al.* reported that 4-methoxy-4′-methylchalcone (3) exhibited strong inhibitory activity against two dermatophytes fungi *Microsporum canis* C112 and *Epidermophyton floccosum* C114 with the MIC value of 3 μg mL^−1^ and 0.5 μg mL^−1^, respectively. In contrast, antifungal activity against *C. albicans*, *Saccharomyces cerevisiae*, *Cryptococcus neoformans*, *Aspergillus niger*, *A. fumigatus* and *A. flavus* was not observed.^31^ In the case of *C. albicans* DSM1386 we noted the same lack of response to the chalcone 3 and also to the other alkyl (methyl and ethyl) derivatives (1, 2, 5). Partial growth inhibition in the presence of 4-ethoxy-4′-methylchalcone was identified. Furthermore, 2,4-dimethoxy-4′-methylchalcone limited the proliferation of *C. albicans* DSM1386 (ΔOD = 0.63). As a result of 4-carboxy-4′-methylchalcone action, high inhibition of *C. albicans* DSM1386 (ΔOD = 0.29) and *F. linii* KB-F1 (ΔOD = 0.32) growth was observed. Moreover, this derivative provided total inhibition of growth of *A. alternata* CBS1526 and *A. niger* DSM1957.

In the literature, there is no information about biological activity of compounds 1a–8a and 8b. As a result of our investigation, all dihydrochalcones (1a–8a) hindered growth of *E. coli* ATCC10536 and prolonged its lag phase. Furthermore, 3-(4-carboxyphenyl)-1-(4-methylphenyl)propan-1-ol (8b) prevented growth of this bacterial strain. In the case of *S. aureus* DSM799 growth, dihydrochalcones with alkyl group attached to the C-4 (1a, 2a, 5a) had inhibitory effect 3 times stronger than corresponding chalcones (1, 2, 5). Furthermore, biotransformation products with *O*-alkyl chains at the C-4 position (3a, 6a) exhibited 2 times stronger activity in comparison to their chalcones (3, 6). Only, 2,4-dimethoxy-4′-methyl-α,β-dihydrochalcone (4a) showed inhibitory effect similar to its chalcone (4). Moreover, 3-(4-carboxyphenyl)-1-(4-methylphenyl)propan-1-ol (8b) exhibited the same strength of action but shortened the lag phase from 28.5 to 10.5 hours in comparison to its chalcone (8). In addition, dihydrochalcone with carboxyl group attached to the C-4 position showed 2 times stronger inhibitory activity than the corresponding chalcone (8) and alcohol (8b). In the case of *C. albicans* DSM1386, alkyl, *O*-alkyl and nitro dihydrochalcone derivatives (1a–3a, 5a–7a) showed antifungal activity about 3 times stronger than the chalcones (1–3, 5–7). Compounds 4a and 8a demonstrated the same influence on growth of this strain, but alcohol 8b exhibited weaker effect of action in comparison to dihydrochalcone 8a. For 3-(4-carboxyphenyl)-1-(4-methylphenyl)propan-1-ol (8b) and other biotransformation products, antifungal properties tested on *A. alternata* CBS1526, *F. linii* KB-F1 and *A. niger* DSM1957 were the same or characterised by reduced activity contrary to starting compounds. Complete inhibition of *A. niger* DSM1957 growth was observed only in the presence of 4-carboxy-4′-methyl-α,β-dihydrochalcone (8a) (ΔOD = 0). Our results showed, chalcones demonstrate high inhibitory activity against Gram-negative strain *E. coli* ATCC10536 in contrast to dihydrochalcones, that hindered the growth of Gram-positive strain – *S. aureus* DSM799.

## Conclusions

Biotransformations of chalcones in *Gordonia* sp. DSM44456 and *Rhodococcus* sp. DSM364 cultures led to corresponding dihydrochalcones. Prolongation of microbial transformation time afforded the product of reduction of carbonyl group – the alcohol. As a result of our investigations, 8 dihydrochalcones and the alcohol were obtained with isolated yields 38–99%. Compounds 2,4-dimethoxy-4′-methyl-α,β-dihydrochalcone (4a), 4-ethyl-4′-methyl-α,β-dihydrochalcone (5a), 4-carboxy-4′-methyl-α,β-dihydrochalcone (8a) and 3-(4-carboxyphenyl)-1-(4-methylphenyl)propan-1-ol (8b) have not been described in known literature so far.

Evaluation of antimicrobial activity allowed us to compare biological properties of synthesised compounds with their corresponding derivatives achieved as a result of microbial transformation. Chalcones 1–6, 8 and novel alcohol 8b exhibited the highest inhibitory effect against *E. coli* ATCC10536 (ΔOD = 0). In the case of *S. aureus* DSM799, dihydrochalcones showed 3 times stronger inhibitory activity than corresponding chalcones. Also, dihydrochalcones expressed higher antifungal activity against *C. albicans* DSM1386. In the case of *A. alternata* CBS1526, *F. linii* KB-F1 and *A. niger* DSM1957 chalcones and dihydrochalcones demonstrated similar activity, excluding 4-carboxy-4′-methyl-α,β-dihydrochalcone (8a), which caused complete inhibition of growth *A. niger* DSM1957 (ΔOD = 0).

## Conflicts of interest

The authors declare no conflict of interest.

## Supplementary Material

RA-008-C8RA04669G-s001
