# Supplementary material for: Microbial transformations of 4′-methylchalcones as an efficient method of obtaining novel alcohol and dihydrochalcone derivatives with antimicrobial activity
Source: RSC Adv. 2018 Aug 30;8(53):30379–86. doi: 10.1039/c8ra04669g (PMC9085419; doi:10.1039/c8ra04669g)
Supplement: RA-008-C8RA04669G-s001 [file RA-008-C8RA04669G-s001.pdf]

Electronic Supplementary Material (ESI) for RSC Advances.
This journal is © The Royal Society of Chemistry 2018

Supplementary Information

Microbial transformations of 4'-methylchalcones as efficient method of obtaining novel alcohol and dihydrochalcone derivatives with antimicrobial activity

Joanna Kozłowska,*[a] Bartłomiej Potaniec,[a] Barbara Żarowska,[b] and Mirosław Anioł[a]

[a] *Department of Chemistry, Wrocław University of Environmental and Life Sciences, Norwida 25, 50-375 Wrocław, Poland. Email: joannakozlowska3@gmail.com,*

[b] *Department of Biotechnology and Food Microbiology, Wrocław University of Environmental and Life Sciences, Chełmońskiego 37, 51-630 Wrocław, Poland*

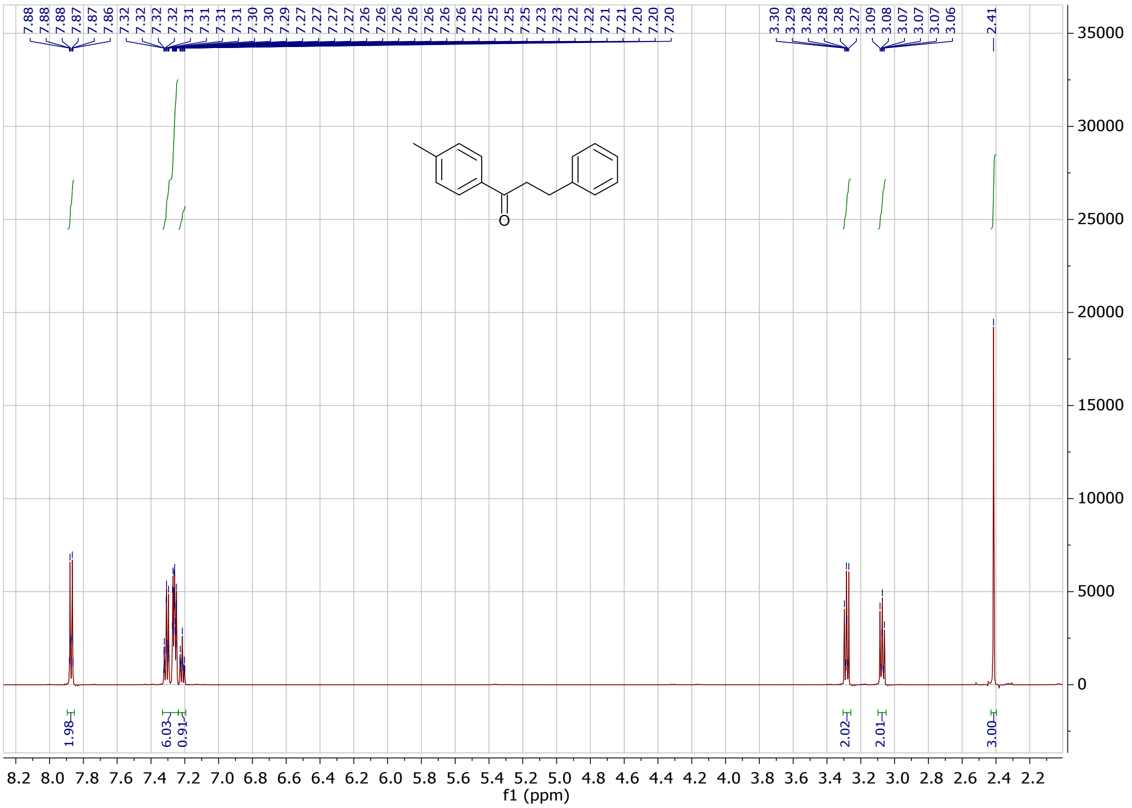

Fig S1. ^{1}H-NMR (600 MHz, $CDCl_3$) spectrum of 4'-methyl-α,β-dihydrochalcone (**1a**)

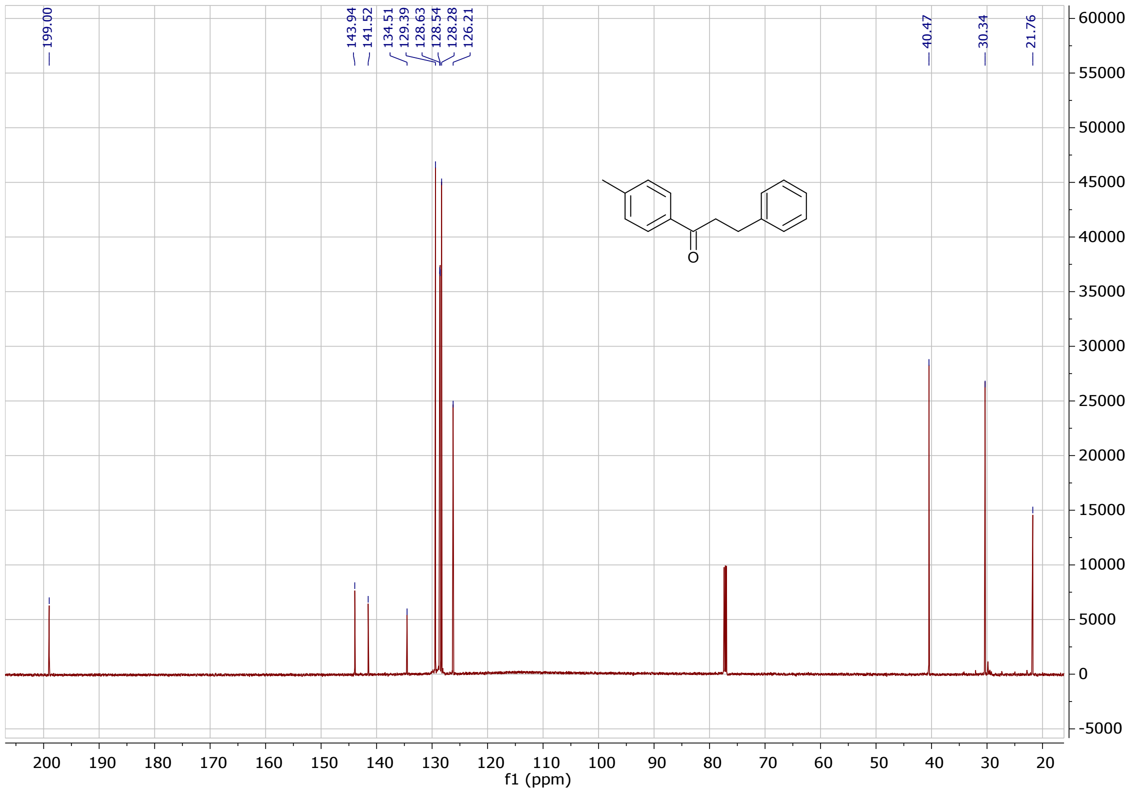

Fig S2. ^{13}C-NMR (150 MHz, $CDCl_3$) spectrum of 4’-methyl-α,β-dihydrochalcone (**1a**)

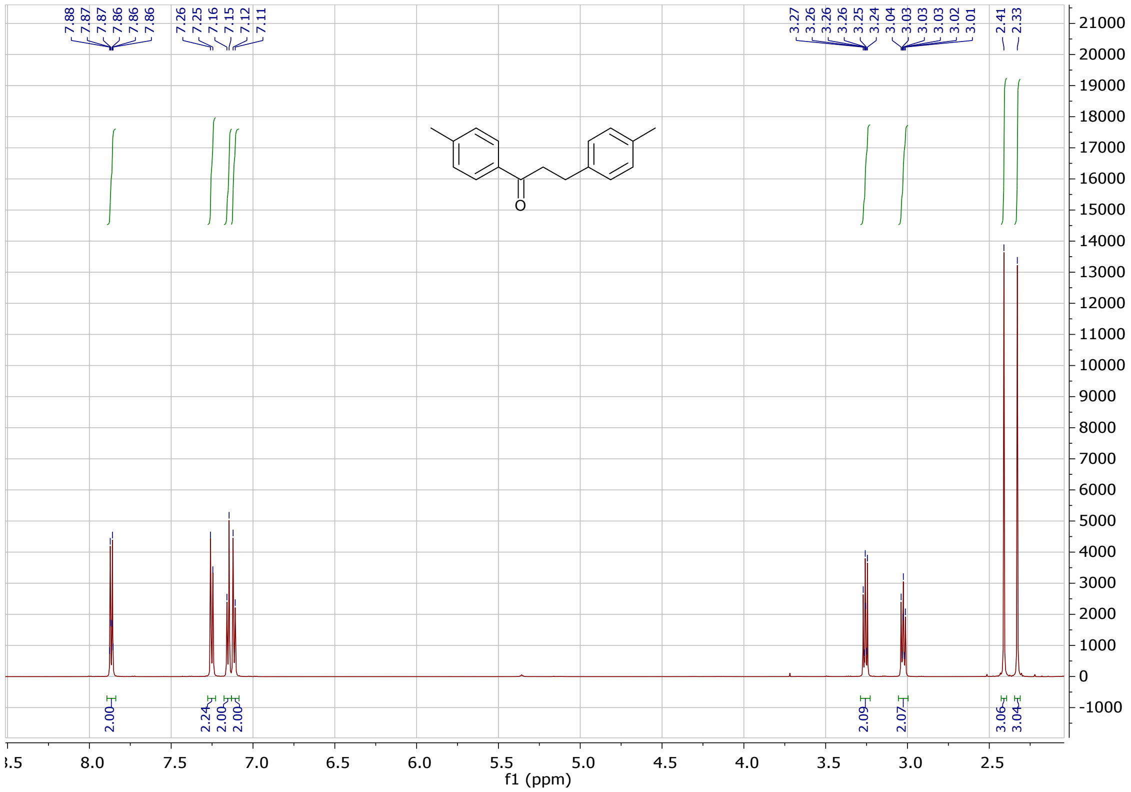

Fig S3. ^{1}H-NMR (600 MHz, $CDCl_3$) spectrum of 4’-methyl-4-methyl-α,β-dihydrochalcone (**2a**)

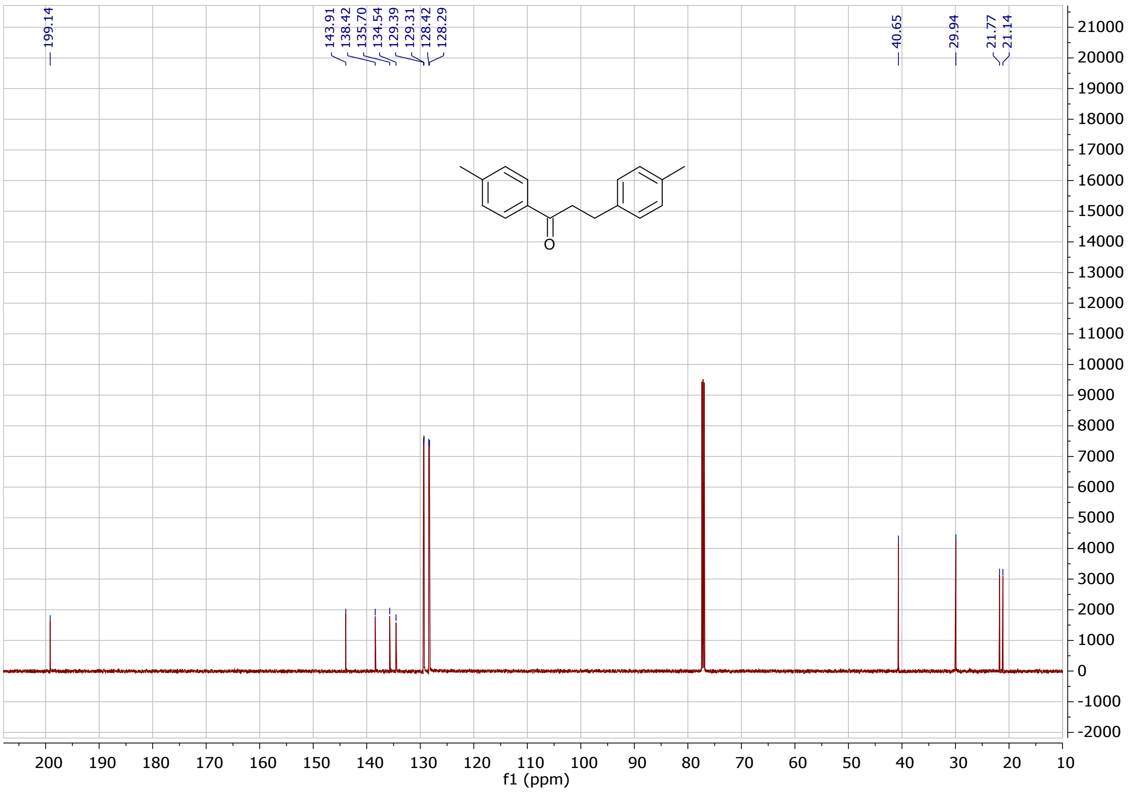

Fig S4. ^{13}C-NMR (150 MHz, $CDCl_3$) spectrum of 4’-methyl-4-methyl-α,β-dihydrochalcone (**2a**)

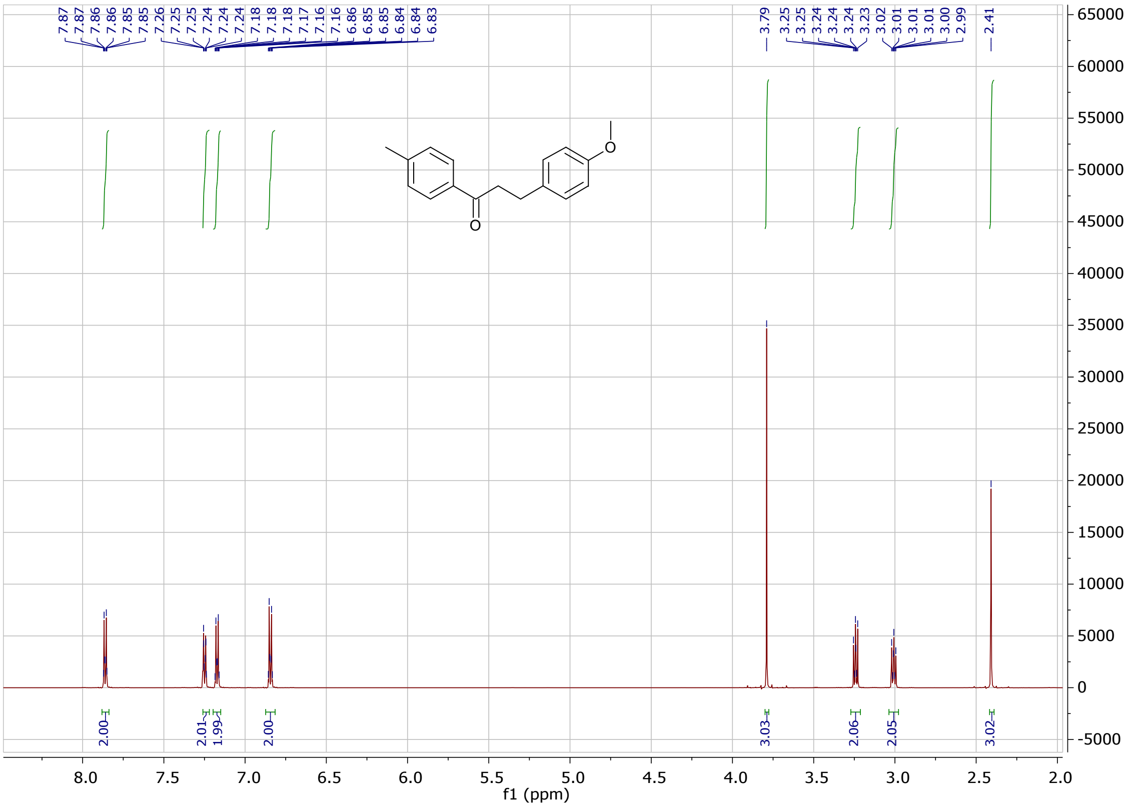

Fig S5. ^{1}H-NMR (600 MHz, $CDCl_3$) spectrum of 4-methoxy-4’-methyl-α,β-dihydrochalcone (**3a**)

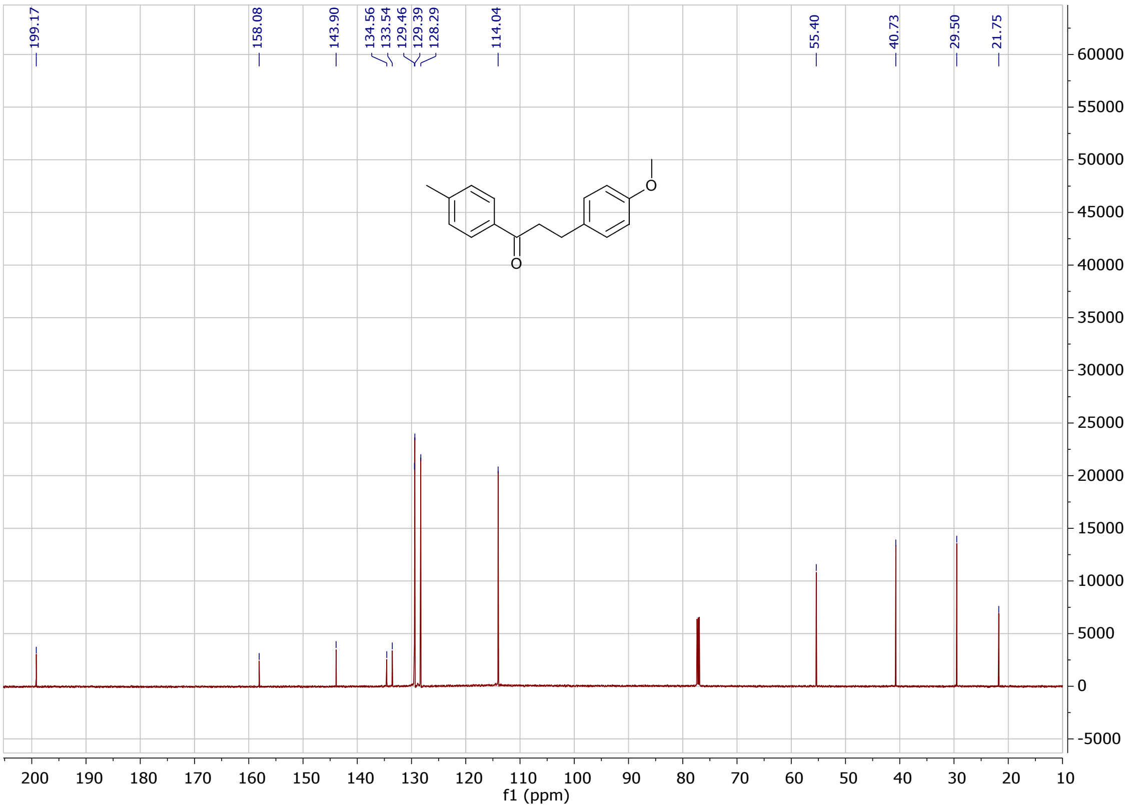

Fig S6. ^{13}C-NMR (150 MHz, $CDCl_3$) spectrum of 4-methoxy-4’-methyl-α,β-dihydrochalcone (**3a**)

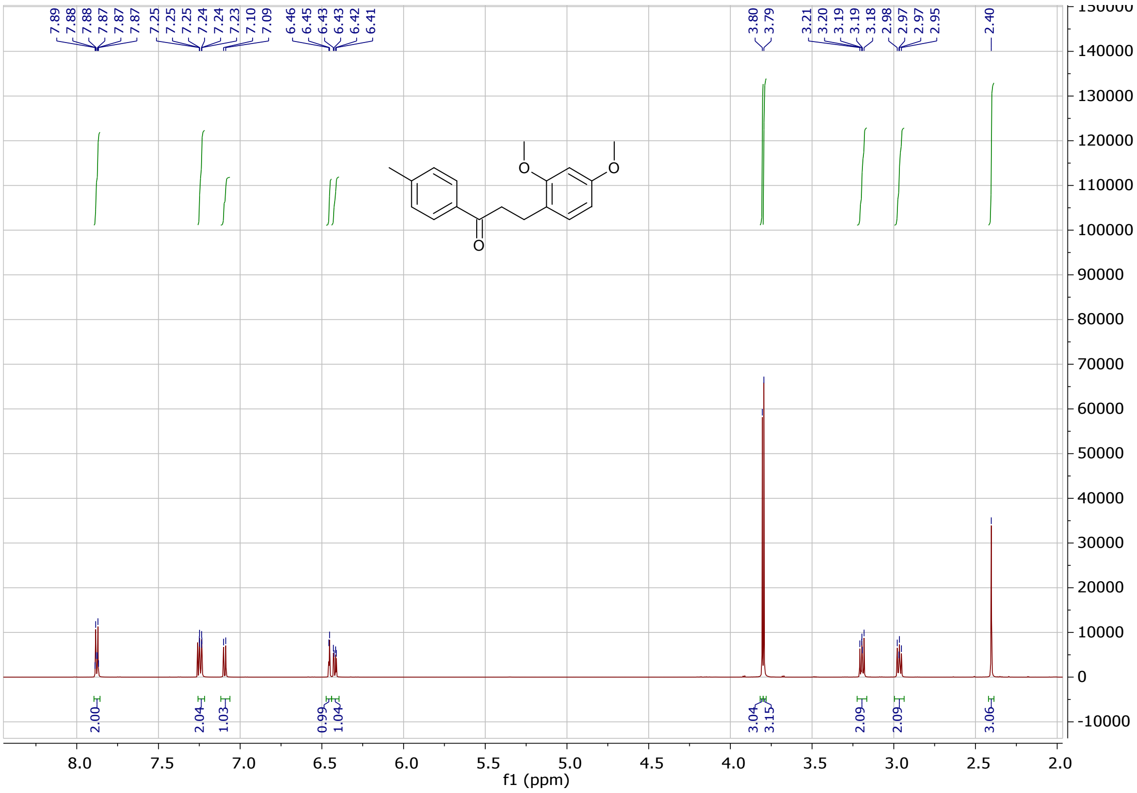

Fig S7. ^{1}H-NMR (600 MHz, $CDCl_3$) spectrum of 2,4-dimethoxy-4’-methyl-α,β-dihydrochalcone (**4a**)

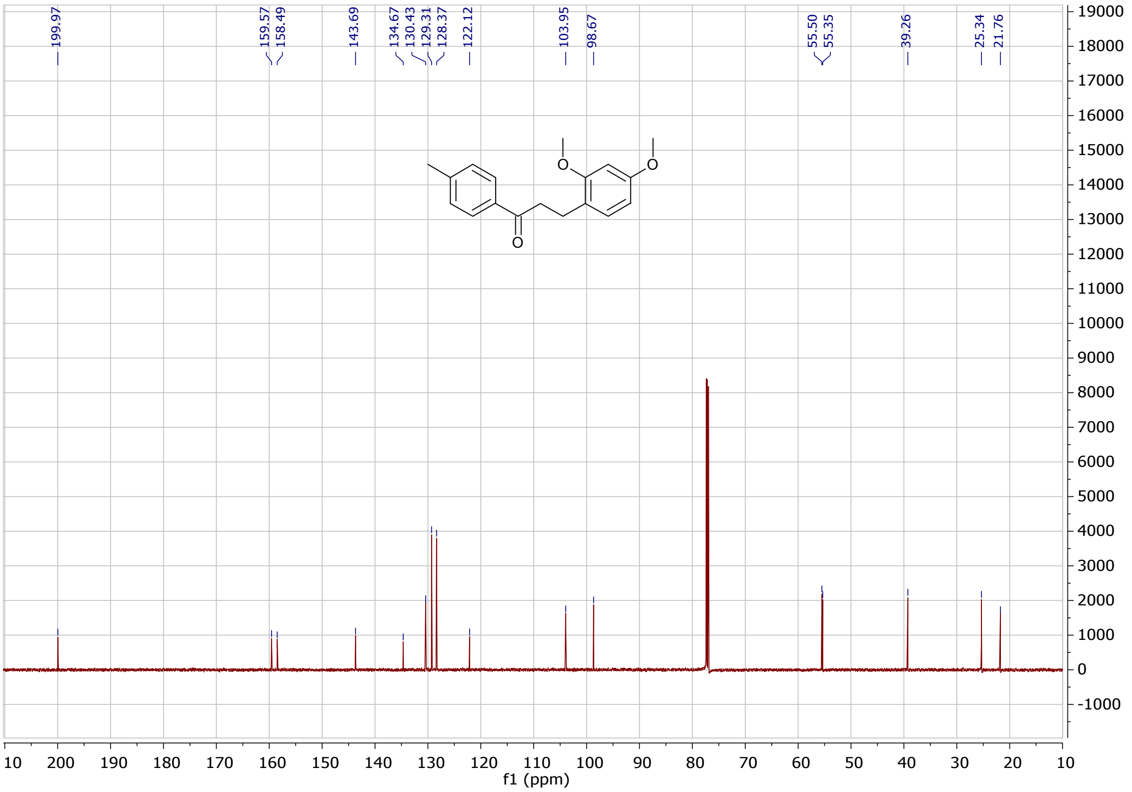

Fig S8. ^{13}C-NMR (150 MHz, $CDCl_3$) spectrum of 2,4-dimethoxy-4'-methyl-α,β-dihydrochalcone (**4a**)

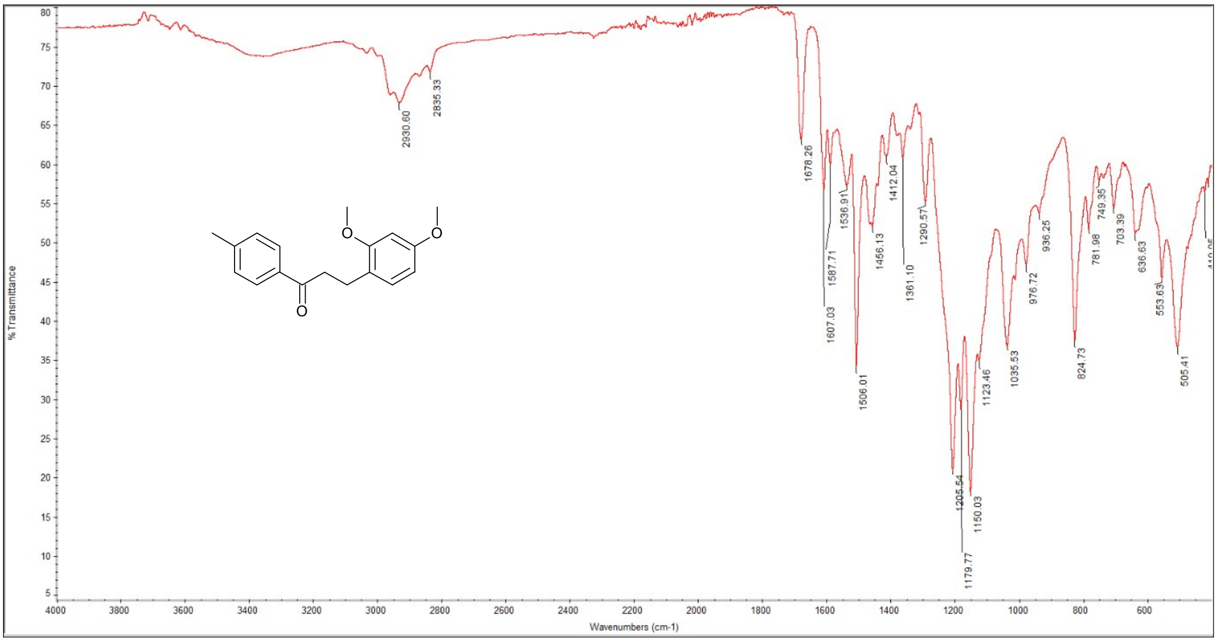

Fig S9. FTIR-ATR spectrum of 2,4-dimethoxy-4'-methyl-α,β-dihydrochalcone (**4a**)

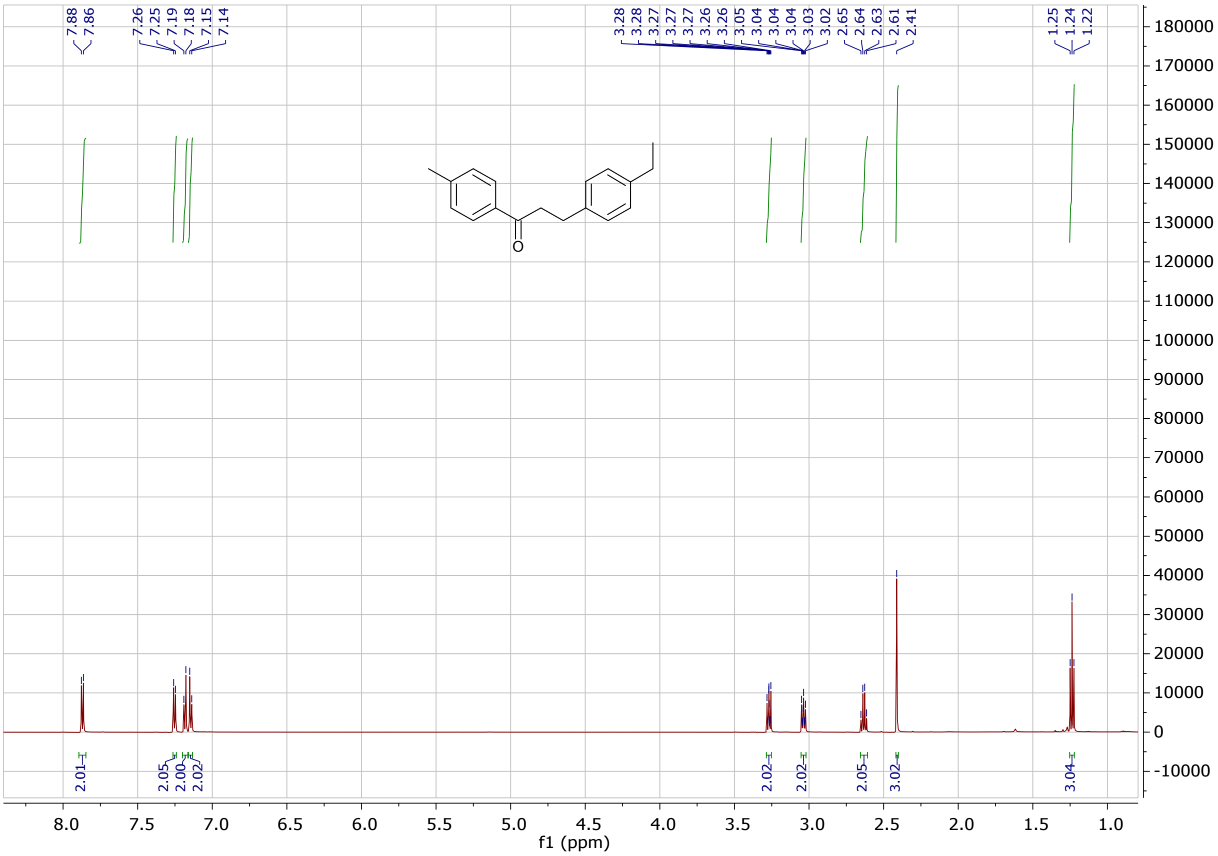

Fig S10. ^{1}H-NMR (600 MHz, $CDCl_3$) spectrum of 4-ethyl-4'-methyl-α,β-dihydrochalcone (**5a**)

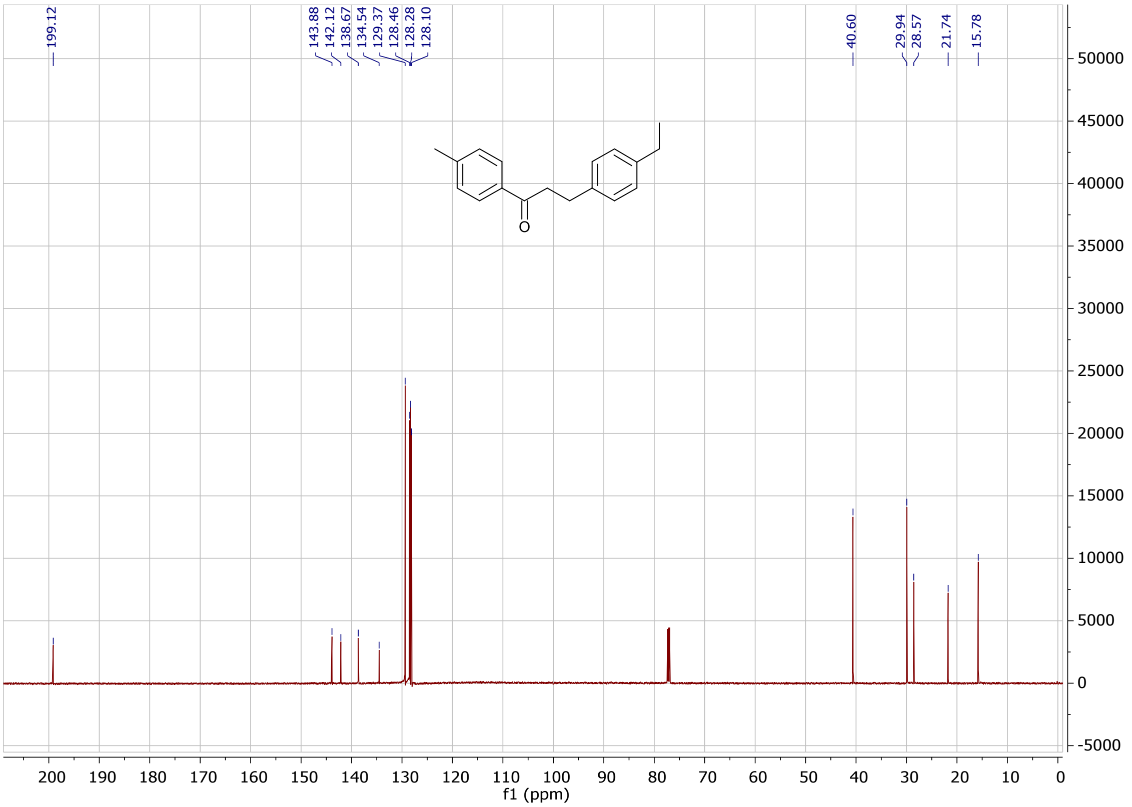

Fig S11. ^{13}C-NMR (150 MHz, $CDCl_3$) spectrum of 4-ethyl-4'-methyl-α,β-dihydrochalcone (**5a**)

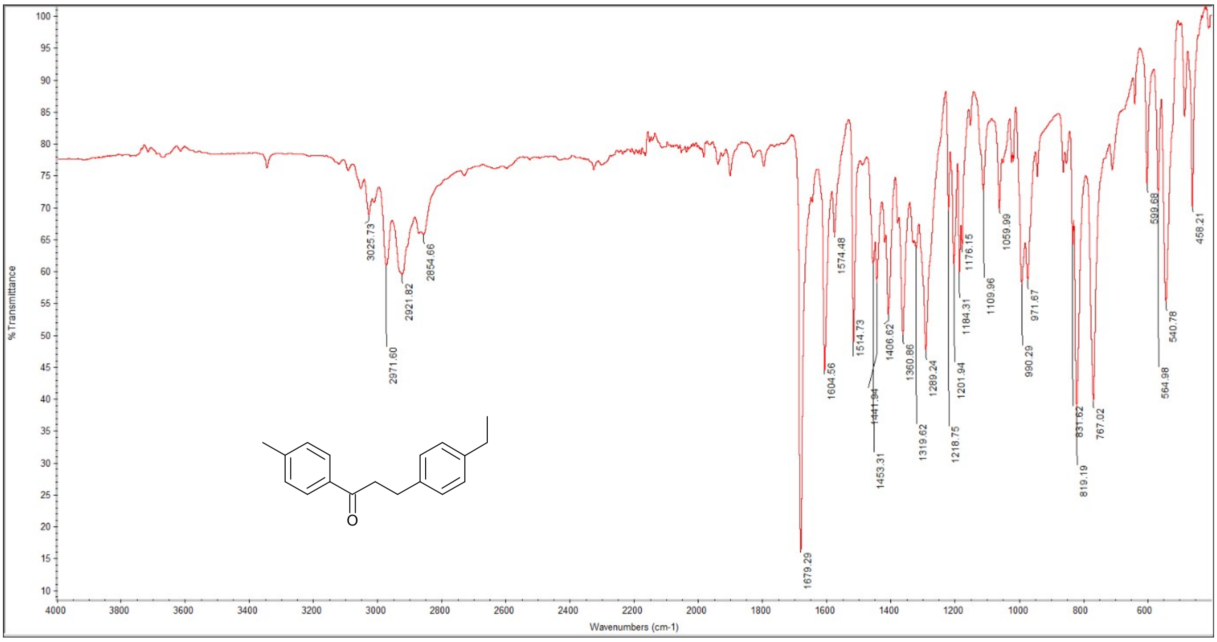

Fig S12. FTIR-ATR spectrum of 4-ethyl-4’-methyl-α,β-dihydrochalcone (**5a**)

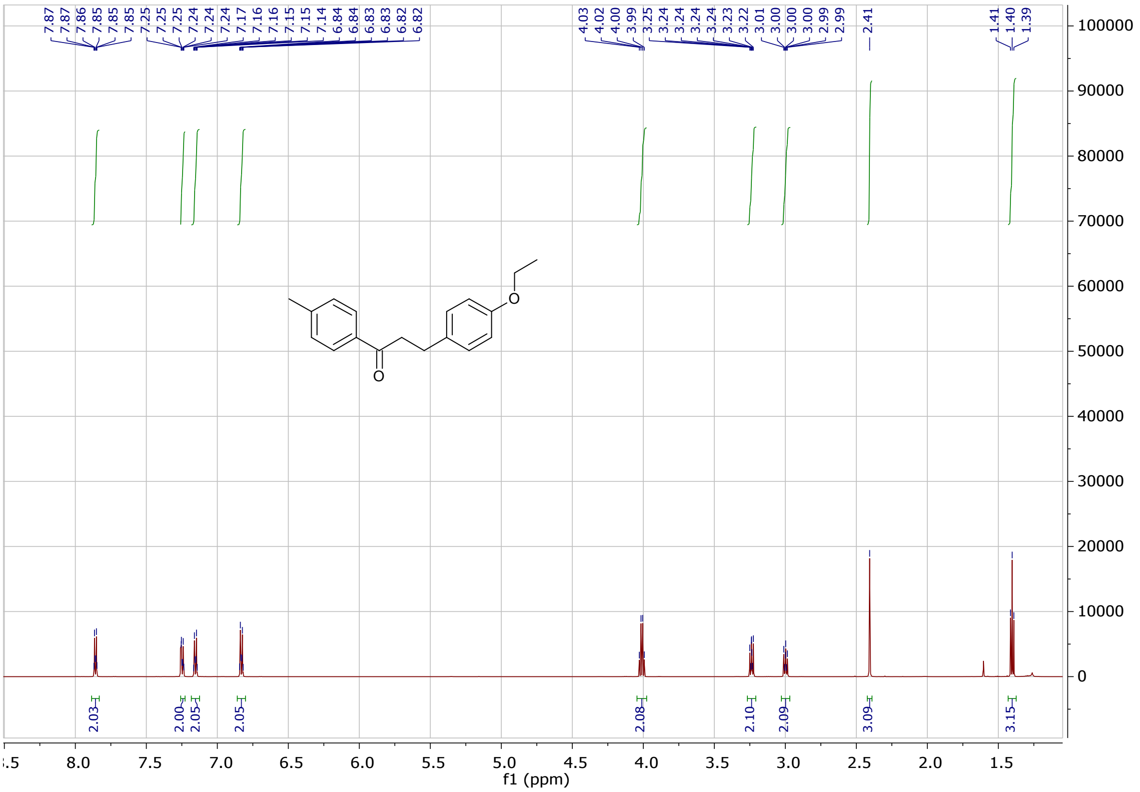

Fig S13. ^{1}H-NMR (600 MHz, $CDCl_3$) spectrum of 4-ethoxy-4’-methyl-α,β-dihydrochalcone (**6a**)

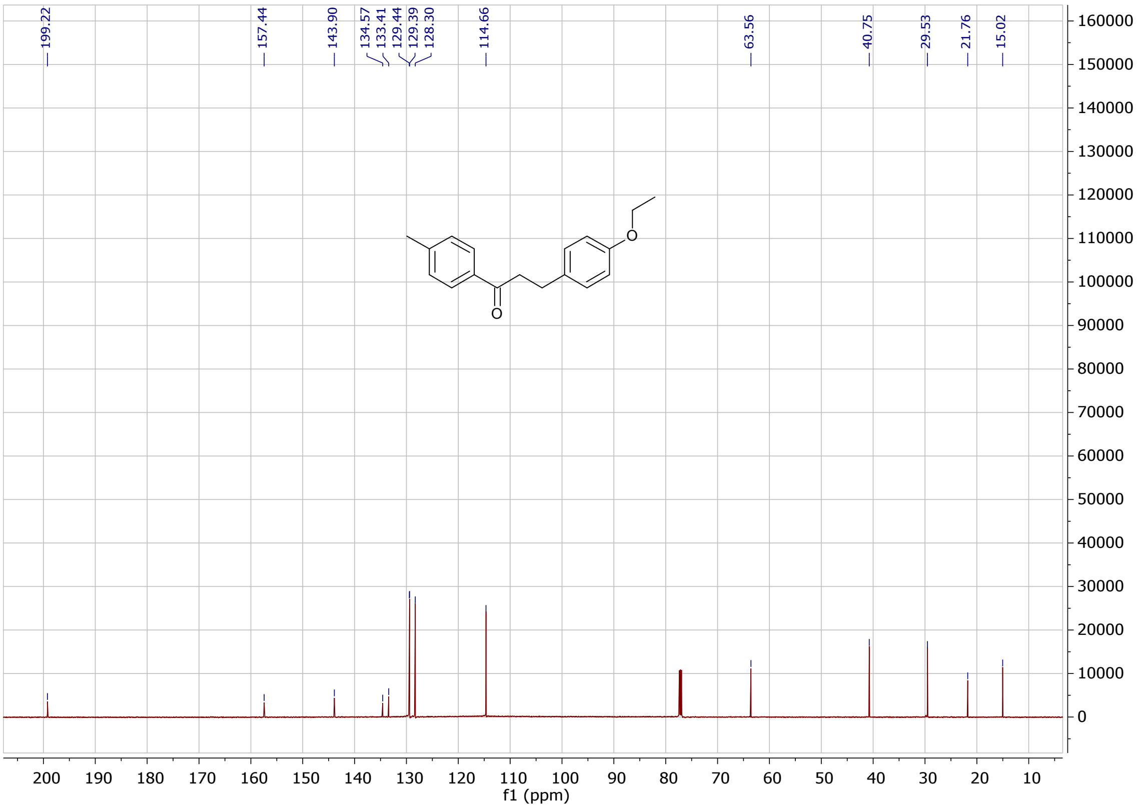

Fig S14. ^{13}C-NMR (150 MHz, $CDCl_3$) spectrum of 4-ethoxy-4'-methyl-α,β-dihydrochalcone (**6a**)

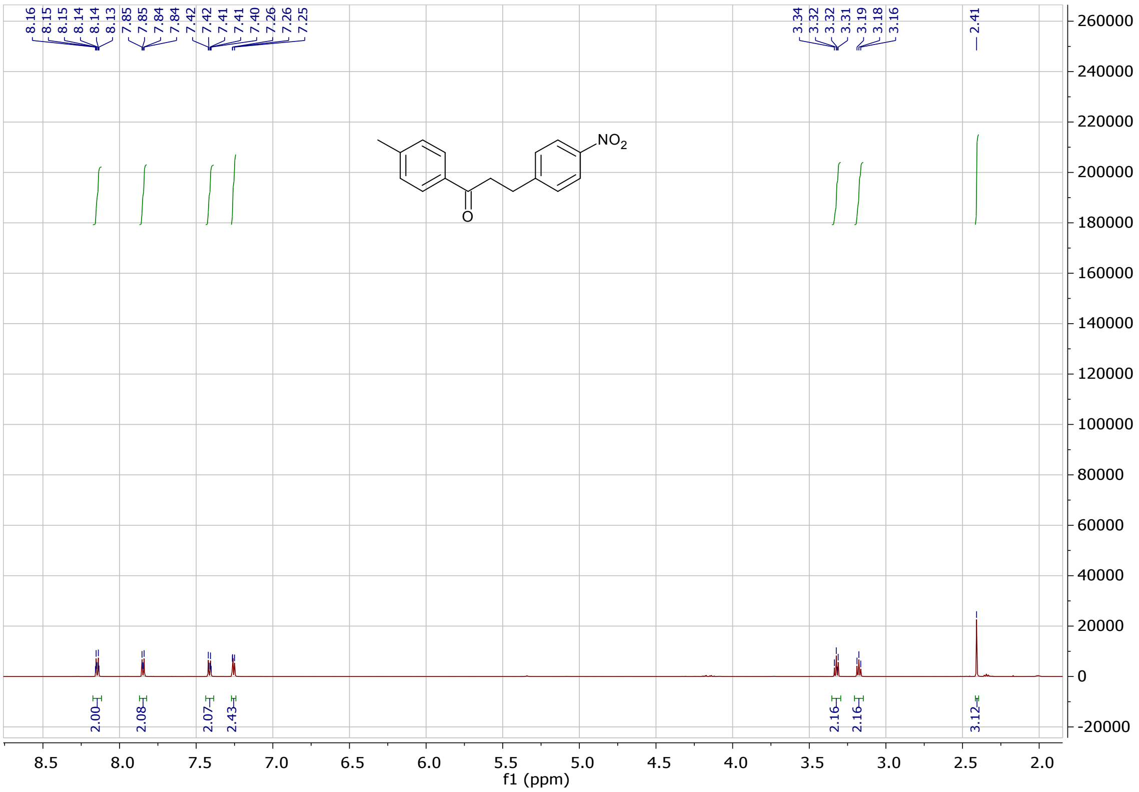

Fig S15. ^{1}H-NMR (600 MHz, $CDCl_3$) spectrum of 4'-methyl-4-nitro-α,β-dihydrochalcone (**7a**)

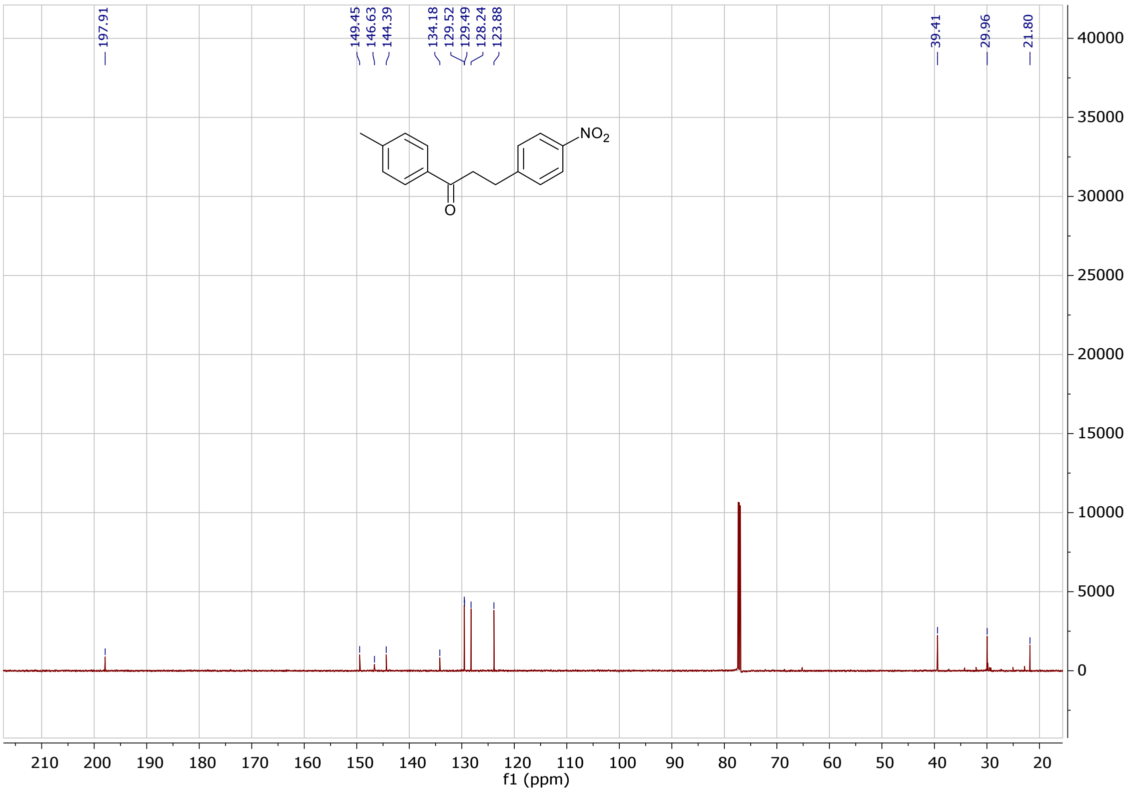

Fig S16. ^{13}C-NMR (150 MHz, $CDCl_3$) spectrum of 4'-methyl-4-nitro-α,β-dihydrochalcone (**7a**)

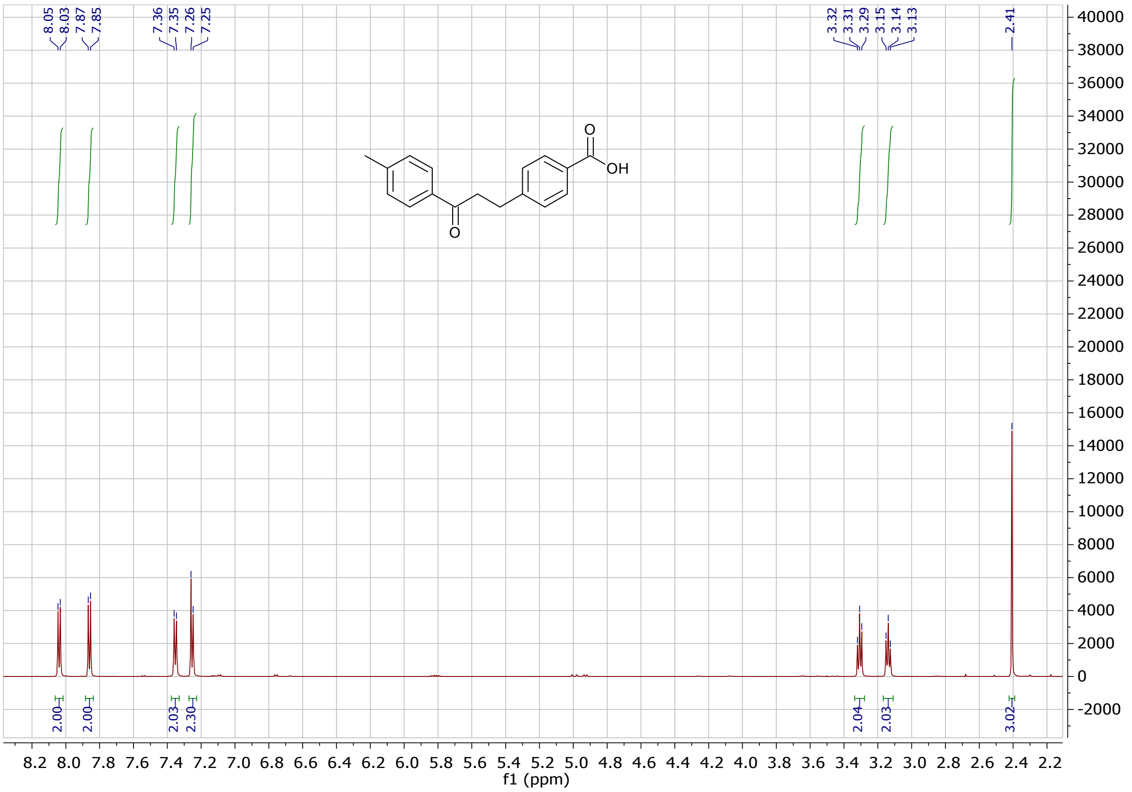

Fig S17. ^{1}H-NMR (600 MHz, $CDCl_3$) spectrum of 4-carboxy-4'-methyl-α,β-dihydrochalcone (**8a**)

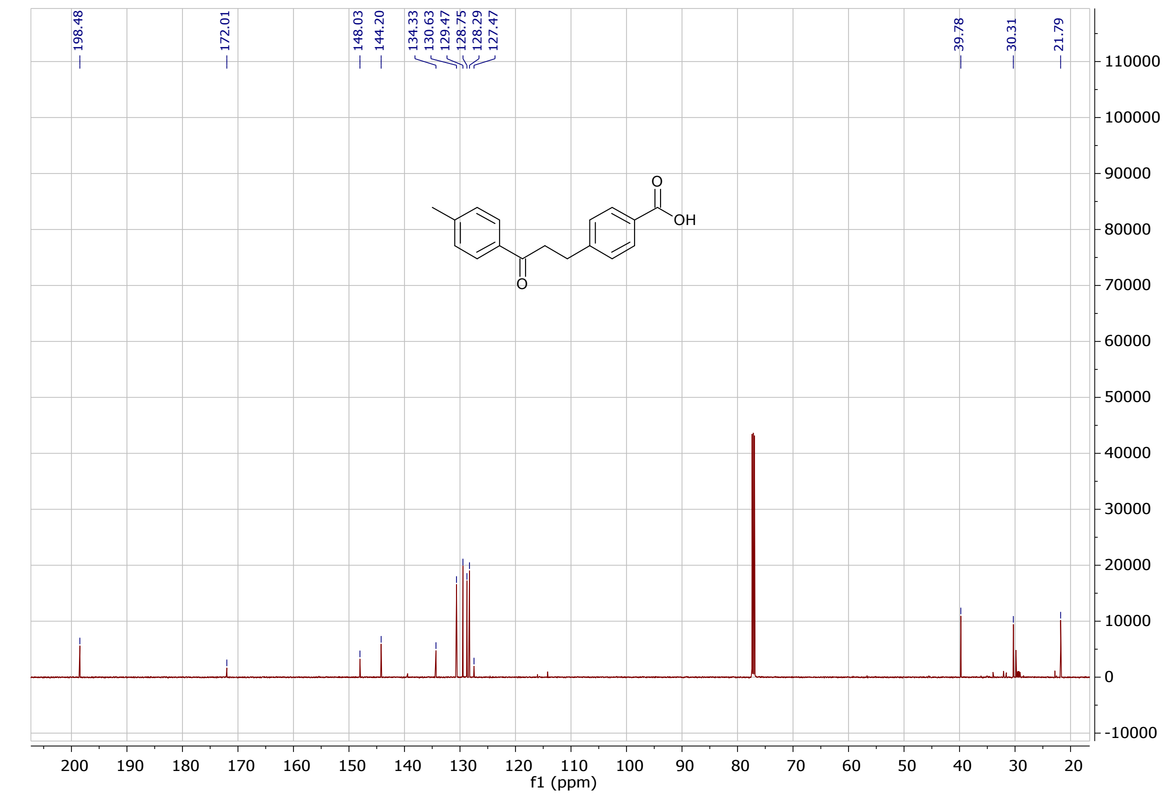

Fig S18. ^{13}C-NMR (150 MHz, $CDCl_3$) spectrum of 4-carboxy-4'-methyl-α,β-dihydrochalcone (**8a**)

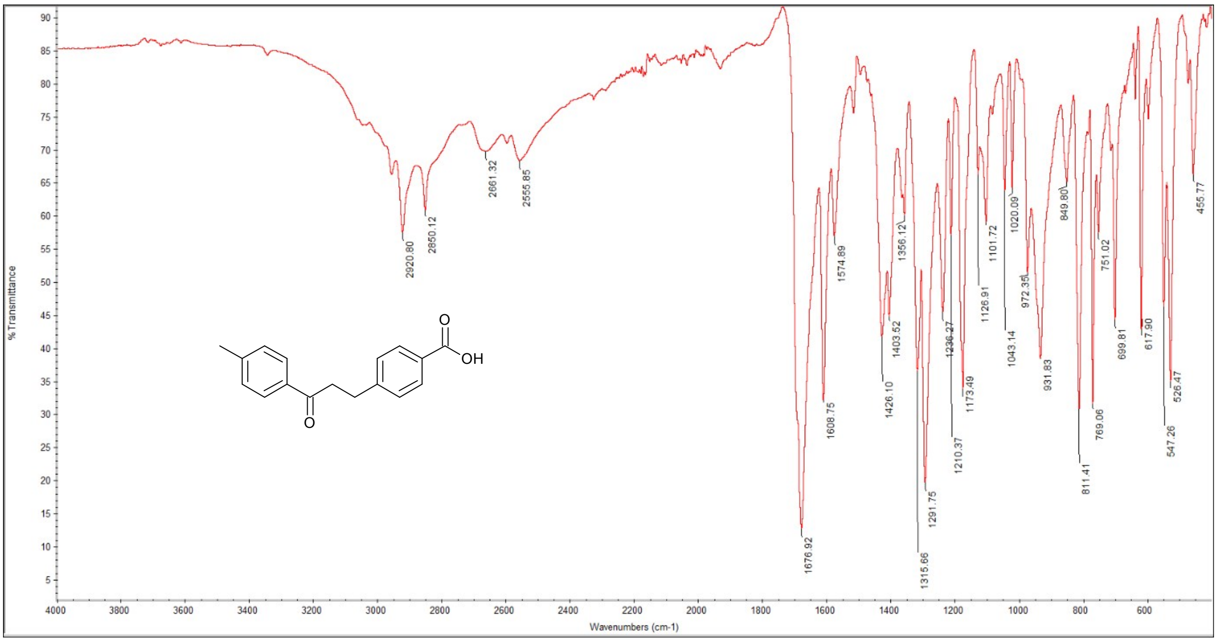

Fig S19. FTIR-ATR spectrum of 4-carboxy-4'-methyl-α,β-dihydrochalcone (**8a**)

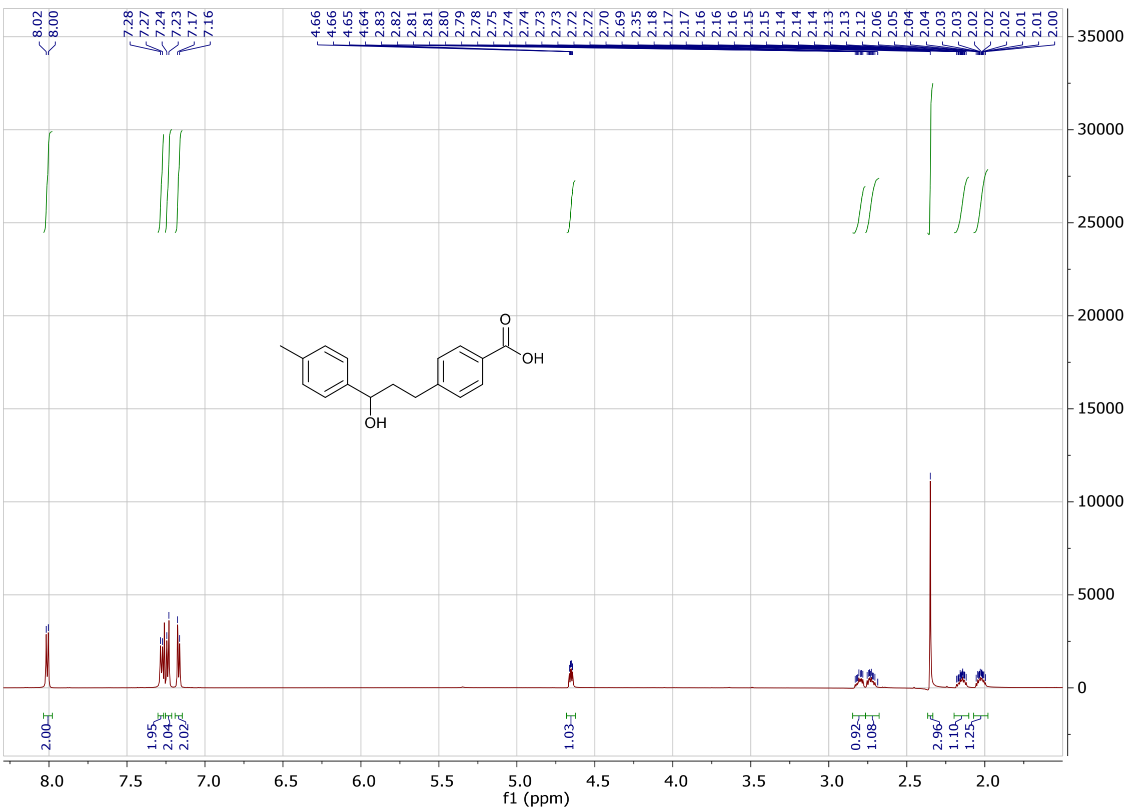

Fig S20. ^{1}H-NMR (600 MHz, $CDCl_3$) spectrum of 3-(4-carboxyphenyl)-1-(4-methylphenyl)propan-1-ol (**8b**)

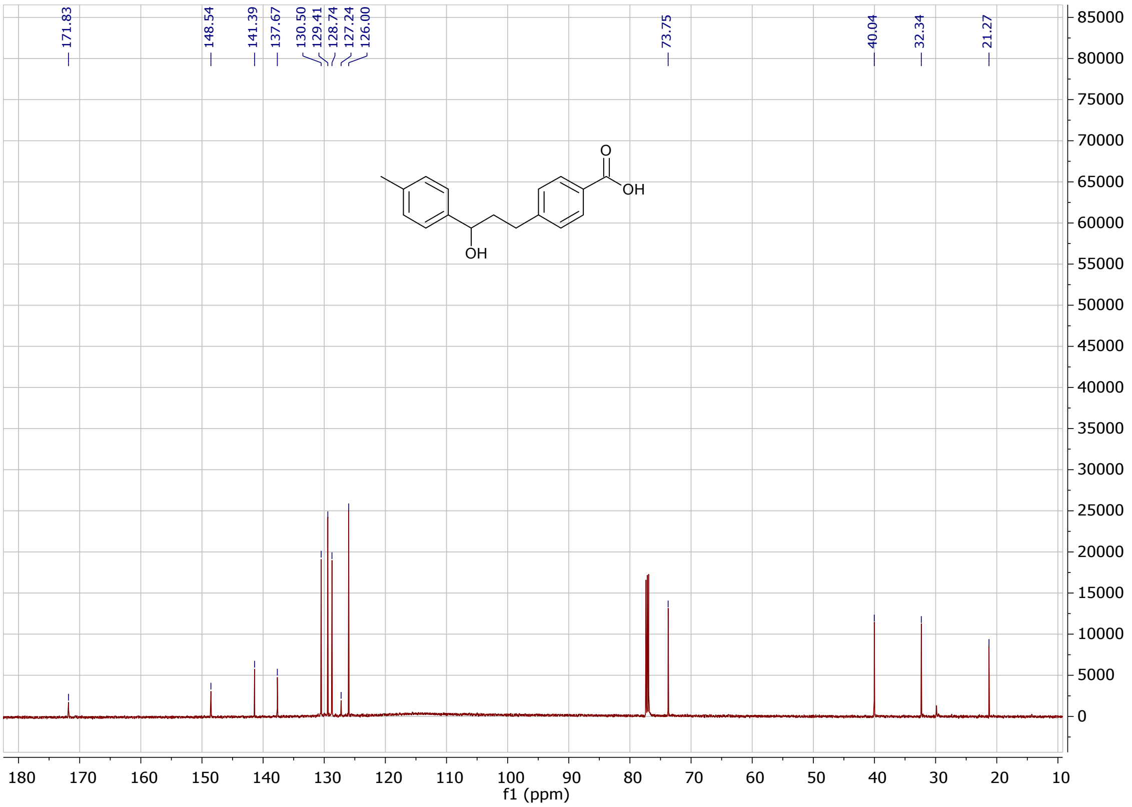

Fig S21. ^{13}C-NMR (150 MHz, $CDCl_3$) spectrum of 3-(4-carboxyphenyl)-1-(4-methylphenyl)propan-1-ol (**8b**)

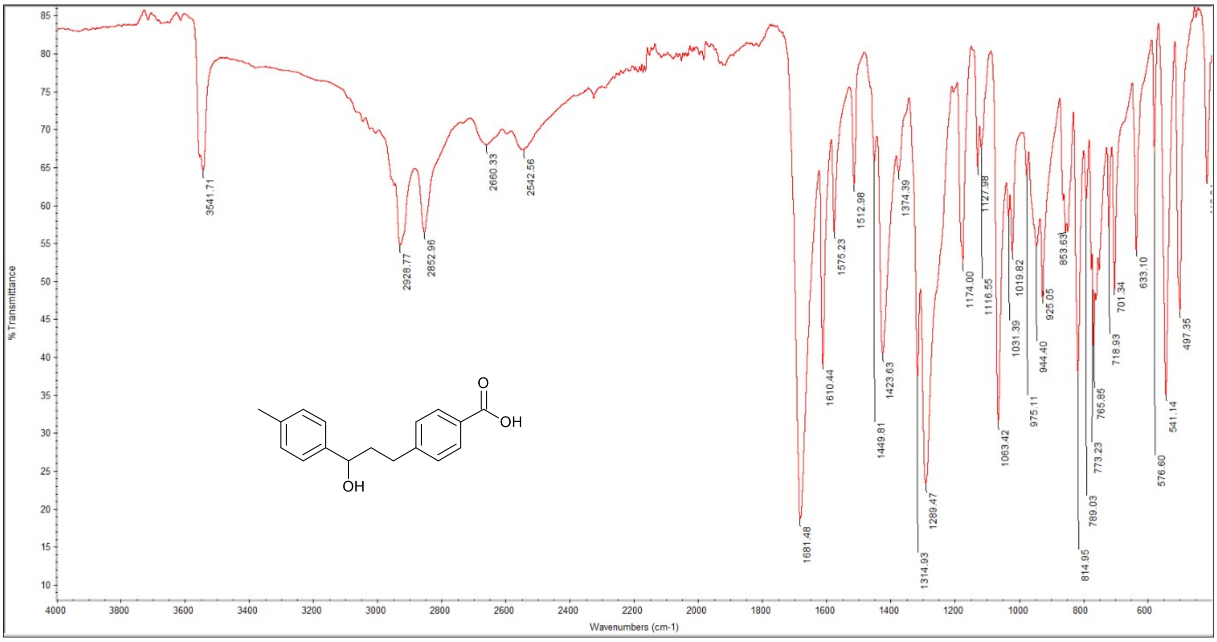

Fig S22. FTIR-ATR spectrum of 3-(4-carboxyphenyl)-1-(4-methylphenyl)propan-1-ol (**8b**)
